# Efficacy of videoconferencing-delivered cognitive behavioural therapy to reduce anxiety disorder severity in LGBTQ+ people: An exploratory trial protocol

**DOI:** 10.1371/journal.pone.0316857

**Published:** 2025-01-24

**Authors:** Isaac B. J. M. D. Dunn, Emma Power, Liam J. Casey, Bethany M. Wootton

**Affiliations:** 1 Discipline of Clinical Psychology, Graduate School of Health, University of Technology, Sydney, New South Wales, Australia; 2 Department of Speech Pathology, Graduate School of Health, University of Technology, Sydney, New South Wales, Australia; Mahidol University, THAILAND

## Abstract

**Objective:**

Cognitive behavior therapy (CBT) is a well-established treatment for anxiety disorders in the general population. However, the efficacy of CBT for lesbian, gay, bisexual, transgender, queer, questioning, and otherwise non-heterosexual or non-cisgender (LGBTQ+) people with anxiety disorders is still emerging in the literature. This protocol proposes an exploratory, two-group, randomized controlled trial comparing the efficacy of CBT for anxiety disorders against a waitlist control group.

**Methods:**

The trial will recruit 52 LGBTQ+ adults with a primary anxiety disorder diagnosis. The treatment will consist of videoconferencing-delivered CBT using the Unified Protocol (UP). The treatment will be provided in eight weekly individual sessions. Following treatment completion, the waitlist control participants will receive an LGBTQ+ adapted CBT intervention delivered via videoconferencing. The control group will receive the LGBTQ+ adapted UP in weekly sessions for eight weeks. Diagnostic status and symptom severity will be assessed at baseline, post-treatment, and three-month follow-up. Post-treatment qualitative exit interviews will collect participant perspectives on treatment acceptability.

**Results:**

Outcome measures will be compared across groups and benchmarked with existing literature to assess efficacy and feasibility, while qualitative analysis will explore intervention acceptability.

**Conclusion:**

The results are anticipated to inform best-practice remote transdiagnostic treatment of anxiety disorders in LGBTQ+ people.

## Introduction

It is estimated that up to 30% of adults will develop an anxiety disorder in their lifetime [1]. The anxiety disorders currently listed in the Diagnostic and Statistical Manual for Mental Disorders (DSM-5-TR) include generalized anxiety disorder, agoraphobia, panic disorder, social anxiety disorder, specific phobia, and separation anxiety disorder [2]. By definition, anxiety disorders cause significant distress or impairment in people’s lives and, without treatment, tend to have a chronic course [2].

For lesbian, gay, bisexual, transgender, queer, questioning, and otherwise non-heterosexual or non-cisgender (LGBTQ+) people, the prevalence of anxiety disorders is elevated when compared to non-LGBTQ+ people [3]. Emerging evidence also suggests that people with variations in sex characteristics, or intersex people, also face high prevalence rates of anxiety disorders [4]. This does not reflect inherent psychopathology in LGBTQ+ people [5, 6]. Rather, a large body of research has indicated that the mechanisms of this disparity involves chronic exposure to stigma and discrimination-based societal threats [5, 6]. Meyer [6] called this chronic experience of excess stress ‘minority stress’.

Minority stressors in Meyer’s model can be understood to be distal, meaning stressors outside of the person, or proximal, meaning stressors within the person [6]. Previous studies have operationalised distal minority stressors as experiences of LGBTQ+ related stigma, prejudice, and discrimination [5, 7]. Proximal minority stressors have previously been operationalised as rejection sensitivity, concealment of identity, and internalized stigma [5, 8, 9], and these stressors have been indicated to be related to anxiety and related disorders [10–12]. Proximal minority stressors may also include more universal behaviours, like rumination or isolation [5, 6]. These can confer risk for developing internalizing disorders, which generally include depressive, obsessive-compulsive, and anxiety and related disorders [13]. While structural change is needed to prevent systemic discrimination issues from affecting mental health outcomes [6], in the meantime, mental health treatments are needed to reduce the higher rates of anxiety disorders that result from minority stressors [14].

Cognitive behavior therapy (CBT) is a first-line psychological therapy for anxiety disorders [15]. This is in part due to meta-analytic evidence indicating that CBT for anxiety disorders has large within-group effect sizes in both clinical trials and effectiveness studies [16]. Due to the frequency of co-occurring anxiety disorders [17], transdiagnostic forms of CBT have been developed to address multiple disorders simultaneously. The Unified Protocol (UP) [18] is one such transdiagnostic form of CBT which has shown promising effiacy in meta-analytic reviews [19]. The UP has shown large within-group and between-group effect sizes for anxiety disorders in the general population when compared to waitlist controls, treatment as usual, and pill placebo control groups [19].

While standard forms of the UP have not yet been tested in LGBTQ+ samples [20–22], LGBTQ+ adapted adaptions of the UP have been developed and examined in LGBTQ+ populations [7–9, 23–25]. The LGBTQ+ adapted UP developed by Pachankis and colleagues [26] still targets universal mechanisms of anxiety and related disorders while taking into account LGBTQ+ people’s minority stress experiences. This is done by including additional therapeutic strategies such as minority stress psychoeducation, assertiveness training, and targeting proximal minority stressors in treatment [27]. Proximal minority stressors are understood to perpetuate the development of internalizing disorders in LGBTQ+ people above and beyond the universal risk factors for anxiety disorder development in the general population [5, 6].

However, to date, few studies examining the LGBTQ+ adapted UP have included participants with diagnosed primary anxiety disorders [7–9, 23, 24]. To date, only one study of gay and bisexual men has included participants with diagnosed anxiety disorders [24]. However, these participants may also have been diagnosed with a primary alcohol or substance use disorder, trauma orstressor related disorder, or depressive disorder, along with self-reported behaviours conferring high risk of HIV transmission [24]. Thus, the efficacy of the LGBTQ+ adapted UP has limited evidence in non-male identified LGBTQ+ people with a diagnosed primary anxiety disorder. Currently, the efficacy of the newer, LGBTQ+ adapted UP when compared to the well-established, standard UP is unknown for LGBTQ+ clients with a primary anxiety disorder.

In exploratory trials, qualitative research offers a wealth of advantageous opportunities beyond quantitative syntheses to both understand and improve intervention trials [28]. For example, qualitative interviews can help optimize intervention delivery and trial conduct, facilitate the interpretation of trial outcomes, and allow community perspectives to be both heard and applied ethically in trials [28]. While participant perspectives on the in-person LGBTQ+ adapted UP are available [8, 27], the post-treatment acceptability of the standard UP or videoconferencing-delivered CBT for LGBTQ+ participants remains unknown.

Additionally, effective CBT interventions can now be delivered in a number of ways, including low-intensity (i.e., internet-delivered, computerized, or bibliotherapy-delivered CBT) and high-intensity (i.e., in-person individual or group interventions, videoconferencing-delivered, or telephone-delivered CBT) interventions [29]. Videoconferencing-delivered CBT has many similarities to CBT delivered in-person. It allows for synchronous, rather than asynchronous, communication between clinican and client, and can be delivered in a 50–60-minute sessions rather than in an online course or reading material format.

Videoconferencing-delivered CBT for internalizing disorders has been suggested to have comparable effiacy to in-person treatment in previous reviews [30, 31]. Disorder-specific meta-analyses have found preliminary evidence of similar effect sizes between in-person and video-conferencing delivered CBT for generalized anxiety disorder [32], social anxiety disorder [33], and panic disorder [34, 35]. However, systematic reviews indicate that currently, no high-intensity, remotely delivered cognitive-behavioral treatments have been evaluated for LGBTQ+ people with internalizing disorders [22]. Alternative approaches to treatment delivery are important because access to mental healthcare for an anxiety disorder is often impacted by geographical [36], psychological [36], and LGBTQ+ specific [37] barriers. Healthcare providers who work in the LGBTQ+ community endorse that high-intensity, remotely delivered treatment could address these barriers to treatment access [38], indicating a need for videoconferencing delivered treatments for LGBTQ+ people.

To address the limitations of the existing literature the aims of the current study are to:

1. Examine the efficacy of videoconferencing-delivered standard CBT for LGBTQ+ adults with an anxiety disorder in a randomized controlled trial using a waitlist control group;
2. Examine the efficacy of videoconferencing-delivered LGBTQ+ adapted CBT for LGBTQ+ adults with an anxiety disorder in an uncontrolled open trial;
3. Benchmark the efficacy of the LGBTQ+ adapted CBT intervention with the standard CBT intervention as well as the existing literature on CBT for anxiety disorders; and
4. Examine the acceptability of standard and LGBTQ+ adapted CBT in LGBTQ+ individuals.

## Materials and method

### Trial design

**Fig 1:**
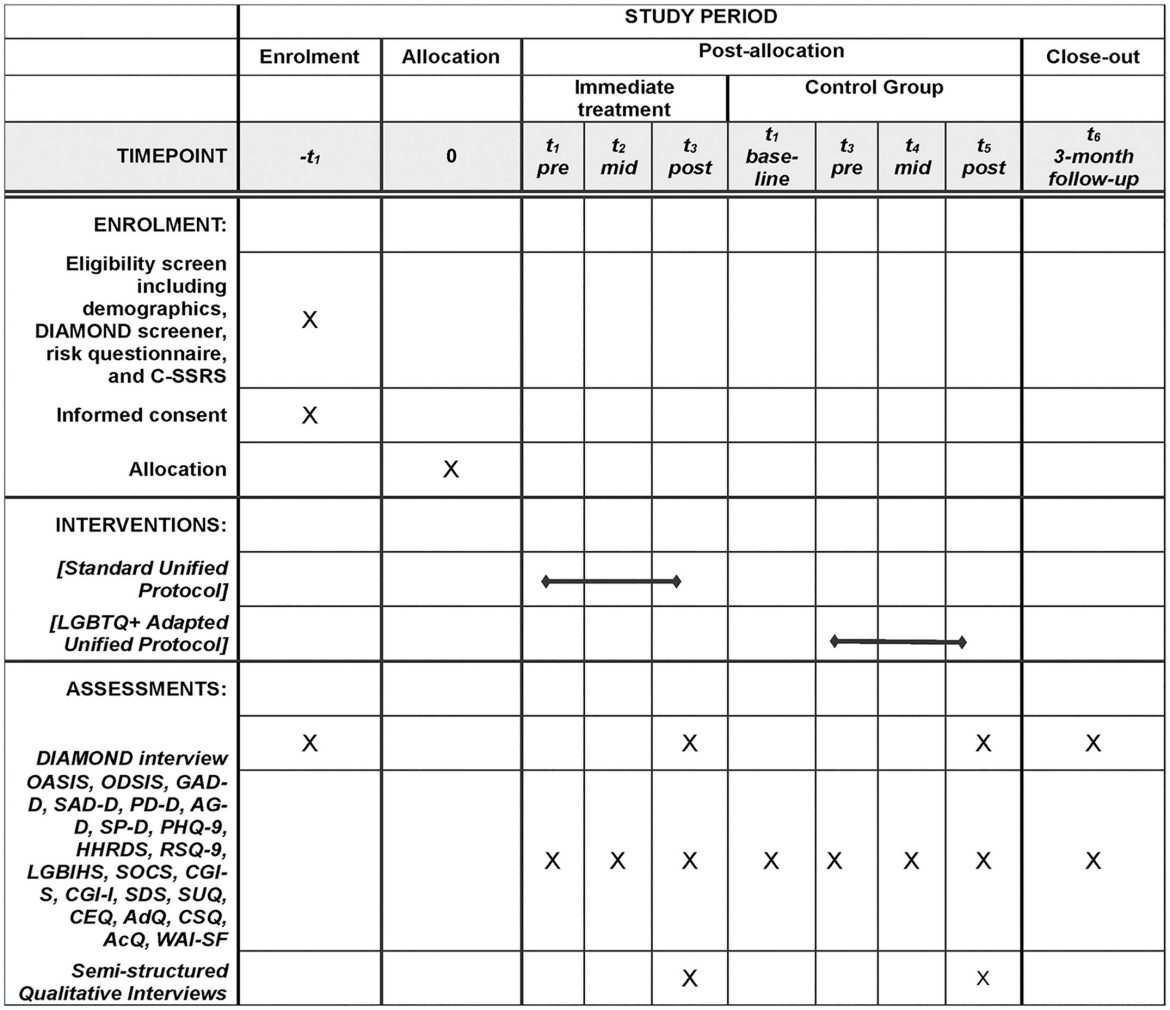
SPIRIT schedule of enrolment.

**Fig 2:**
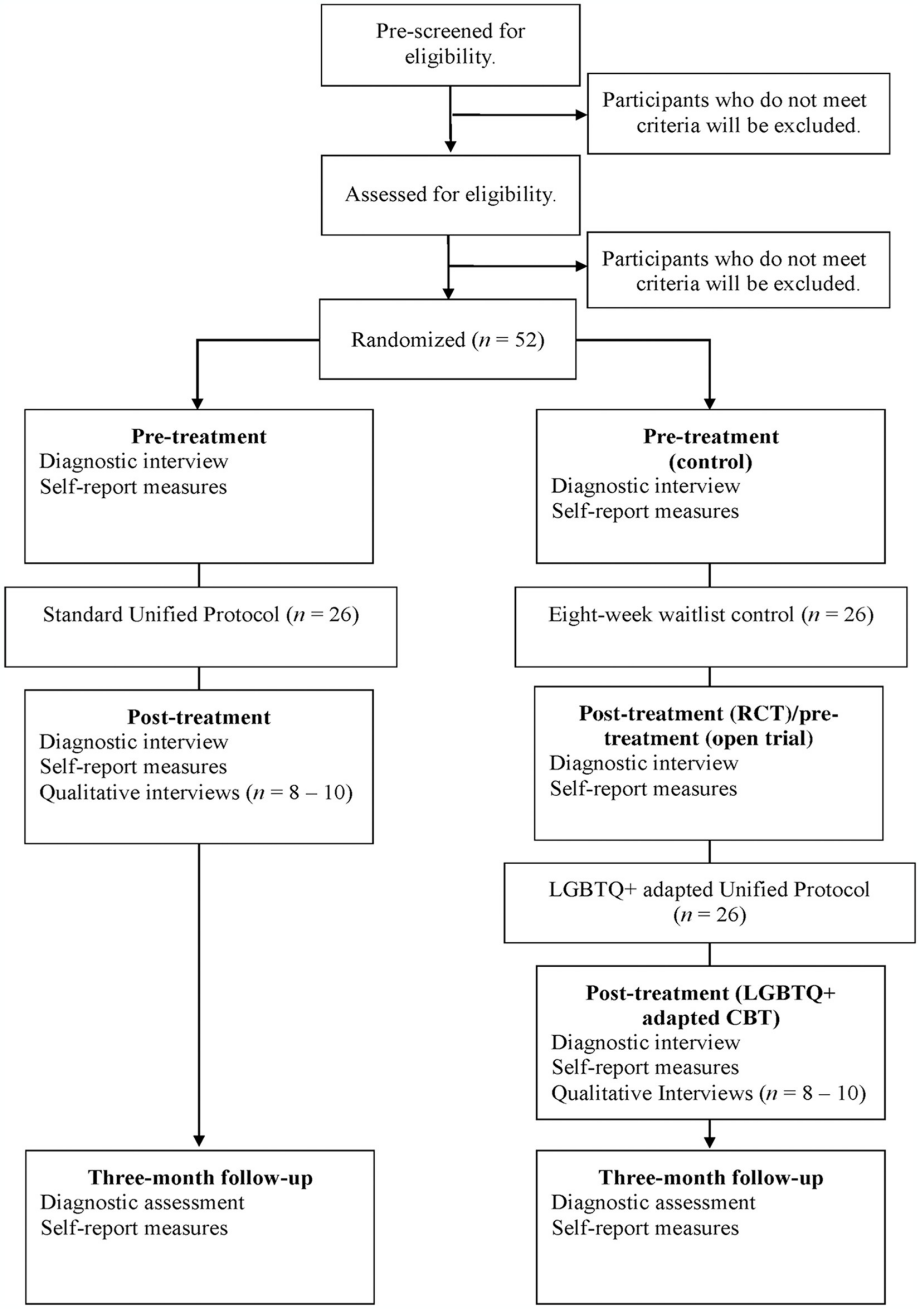
Study flow diagram.

This trial is designed as an exploratory, CONSORT-R compliant, randomized control trial (RCT) with two groups, as seen in Fig 1 [39]. Group 1 is an immediate treatment group (*n* = 26), and Group 2 is a waitlist control group (*n* = 26). A waitlist control group is justified in this instance, given that there have been no studies that have examined the efficacy of the standard UP in LGBTQ+ adults [40] and the aim of the study is to examine the acceptability of this protocol before progressing to larger trials [41]. As such, this is a Phase 1 clinical trial of standard CBT delivered via videoconferencing, with an open trial of LGBTQ+ adapted CBT offered to waitlist participants. The study flow is outlined in Fig 2. The present protocol adhered to SPIRIT [39] and TIDieR [42] reporting guidelines, available in S1 and S2 Tables.

### Ethical approval and trial registration

This trial has been approved by the University of Technology Sydney Health and Medical Research Ethics Committee (UTS MREC; REF NO. ETH23-8902). The trial has been pre-registered with the Australian and New Zealand Clinical Trials Registry (ANZCTR; ACTRN12624000453583). Any unforeseen modifications to the trial will be updated via the ANZCTR registry following relevant ethical approval.

### Participants

Fifty-two participants will be recruited for this exploratory trial. Inclusion and exclusion criteria are outlined in Table 1. For qualitative interviews nested within the trial, 16–20 participants will be recruited.

**Table 1:** Inclusion criteria and associated rationale.

Inclusion Criteria	Rationale
(1) Presently living in Australia	Study target population
(2) Aged 18 years or older	Study target population
(3) Fluent in English	Internal validity confound
(4) Meets criteria for an anxiety disorder as either the primary diagnosis or co-primary diagnosis with a depressive disorder, and the disorder is of at least ’moderate severity’ on the module severity measure of the Diagnostic Interview for Anxiety, Mood, and Obsessive-compulsive and Related Neuropsychiatric Disorders (DIAMOND)	Study target population
(5) Medication-free or on a stable dose (8 weeks) of psychotropic medication	Internal validity confound
(6) Not receiving regular psychological services for anxiety symptoms (defined as at least weekly sessions with a qualified mental health professional)	Internal validity confound
(7) Identifies as LGBTQ+	Study target population
(8) Has access to a private location to complete the treatment for the duration of the study	Feasibility
Exclusion criteria	Rationale
(1) Suicide risk as assessed by item 9 of the PHQ-9 (a score of 2 or above) at baseline, the Columbia-Suicide Severity Rating Scale (C-SSRS), self-report on the risk questionnaire, or via clinician judgment during the diagnostic interview	Participant safety
(2) Engagement in non-suicidal self-injury in the past 12 months	Participant safety
(3) Daily use of alcohol or illicit drugs	Internal validity confound
(4) The presence of a schizophrenia spectrum disorder as assessed by the DIAMOND	Internal validity confound
(5) Any significant cognitive/intellectual impairment as assessed during the diagnostic interview	Internal validity confound
(6) A medical condition that may interfere with treatment	Internal validity confound
(7) No access to a computer with a camera and stable internet on a regular basis	Feasibility
(8) Is not willing to engage in treatment on a regular basis using internet-videoconferencing software	Feasibility
(9) Does not explicitly identify as LGBTQ+	Study target population
(10) Does not indicate any anxiety disorder symptoms on the DIAMOND Self Report screener	Study target population

### Recruitment

Participants will be recruited via social media platforms, professional networking sites, and flyers placed on community noticeboards. Australian health professionals, such as general practitioners, psychiatrists, and psychologists will also be informed of the study via letters and/or email and may refer their clients when appropriate. Advertisement applications will be lodged with not-for-profit organizations to advertise the study where possible, and a link to the Participant Information and Consent Form (PICF) will be available on a website affiliated with the research team. These recruitment methods are consistent with previous trials [8, 24, 27] and recruitment source will be monitored throughout the study. Participants will be required to provide written informed consent via the PICF, available through all recruitment methods, prior to commencing any initial screening measures. Recruitment commenced on 04/06/2024 and is anticipated to continue until the selected sample size has been reached or the trial is ceased.

### Procedure

First, participants who provide online consent will complete initial screening questionnaires online via RedCAP [43, 44]. Then, based on the screening questionnaire, participants likely to meet the inclusion criteria will be invited to attend a diagnostic interview to confirm the study inclusion criteria. Participants who meet entry criteria will be enrolled by assessing clinicians and then randomized by the Principal Investigator (PI) to an eight-week treatment or an eight-week waitlist control group using a random number generator provided via the website www.random.org. Participants in the immediate treatment group will receive eight 50–60-minute sessions of standard, unadapted, transdiagnostic CBT delivered via videoconferencing software Zoom. After the eight-week waitlist period ends, participants in the waitlist control group will receive LGBTQ+ adapted transdiagnostic CBT delivered over eight 50–60-minute sessions via Zoom.

### Measures

The SPIRIT outcome administration schedule is outlined in Table 2. Screening measures are anticipated to take 30 minutes. Outcome measures are anticipated to take about 40 minutes. Diagnostic assessments are anticipated to take approximately 1–2 hours but may take longer depending on the participant’s presenting concerns [45].

**Table 2:** Outcome measures administration schedule.

	Screening	Group 1: Immediate Treatment				Group 2: Control Group				
	Both groups	Pre-(Week 0)	Mid-	Post-(Week 9)	3-month follow-up	Baseline (Week 0)	Pre-(Week 9)	Mid-	Post- (Week 18)	3-month follow-up
**Screening Measures**										
* Demographics*	+									
* Risk Questionnaire*	+									
* DIAMOND Screener*	+									
* DIAMOND Interview*	+			+	+		+		+	+
* C-SSRS*	+									
**Assessments**										
**Primary Outcome Measures**										
* OASIS*		+	+	+	+	+	+	+	+	+
**Secondary Outcome Measures**										
* ODSIS*		+	+	+	+	+	+	+	+	+
* GAD-D*		+	+	+	+	+	+	+	+	+
* SAD-D*		+	+	+	+	+	+	+	+	+
* PD-D*		+	+	+	+	+	+	+	+	+
* AG-D*		+	+	+	+	+	+	+	+	+
* SP-D*		+	+	+	+	+	+	+	+	+
* SepAD-D*		+	+	+	+	+	+	+	+	+
* PHQ-9*	+	+	+	+	+	+	+	+	+	+
* HHRDS*		+	+	+	+	+	+	+	+	+
* RSQ-A*		+	+	+	+	+	+	+	+	+
* LGBIS*		+	+	+	+	+	+	+	+	+
* SOCS*		+	+	+	+	+	+	+	+	+
* CGI-S*	+	+	+	+	+	+	+	+	+	+
* CGI-I*			+	+	+			+	+	+
* SDS*		+	+	+	+	+	+	+	+	+
**Process/Acceptability Measures**										
* SUQ*				+	+				+	+
* CEQ*		+					+			
* AdQ*			+	+	+			+	+	+
* CSQ*			+	+				+	+	
* AcQ*			+	+				+	+	
* WAI-SF*			+	+				+	+	
* Semi-Structured Qualitative Interviews*				+					+	

*Note*. AcQ = Acceptability Questionnaire; AdQ = Adherence Questionnaire; AG-D = Agoraphobia Dimensional Scale; CEQ = Credibility/Expectancy Questionnaire; CGI-S = NIMH Clinician Global Impression Scale–Severity; CGI-I = NIMH Clinician Global Impression Scale–Global Improvement; CSQ = Client Satisfaction Questionnaire; C-SSRS = Columbia-Suicide Severity Rating Scale; DIAMOND = Diagnostic Interview for Anxiety, Mood, and Obsessive-Compulsive and Related Neuropsychiatric Disorders; HHRDS = Heterosexist Harassment, Rejection, and Discrimination Scale; LGBIHS = Lesbian, Gay, Bisexual Identity Scale–Internalized Homophobia Subscale; OASIS = Overall Anxiety Severity and Impairment Scale; ODSIS = Overall Depression Severity and Impairment Scale; PD-D = Panic Disorder Dimensional Scale; RSQ-A = Rejection Sensitivity Questionnaire–Adult; SP-D = Specific Phobia Dimensional Scale; SAD-D = Social Anxiety Disorder-Dimensional Scale; PHQ-9 = Patient Health Questionnaire-9 item; SOCS = Sexual Orientation Concealment Scale; SUQ = Service Use Questionnaire; WAI-SF = Working Alliance Inventory–Short Form.

#### Screening measures

*Demographic questionnaire*. A 22-item demographic questionnaire will collect information on age, location, gender identity, sex assigned at birth, sexual orientation, marital status, employment and education status, English fluency, racial and ethnic self-identification, access to a private location and private computer, and medication use.

*Risk questionnaire*. A five-item risk questionnaire used in prior studies [46] will measure suicide and deliberate self-harm history, regular alcohol and illicit drug use, and suicidal ideation.

*Diagnostic Interview for Anxiety*, *Mood*, *and Obsessive-compulsive and Related Neuropsychiatric Disorders (DIAMOND)* [45]. Diagnostic status will be assessed using the semi-structured Diagnostic Interview for Anxiety, Mood, and Obsessive-Compulsive and Related Neuropsychiatric Disorders (DIAMOND) [45]. The DIAMOND has been shown to have very good test-retest reliability (κ = .75), very good interrater validity (κ = .73) and convergent predictive validity for all anxiety disorders [45]. The DIAMOND includes a 30-item self-report questionnaire, which is used to indicate to the clinician which modules of the DIAMOND need to be administered during the semi-structured diagnostic interview. All assessing clinicians will be registered or provisionally registered psychologists in their final year of the Master of Clinical Psychology program and will be supervised by an experienced clinical psychologist. All assessing clinicians will complete formalized online training on the DIAMOND, and interrater reliability will be assessed. The diagnostic screening will occur via videoconferencing.

*Columbia-Suicide Severity Rating Scale (C-SSRS)*. The severity of suicidal ideation and behavior will be assessed using the semi-structured Columbia-Suicide Severity Rating Scale (C-SSRS) interview [47]. The C-SSRS is a six-item semi-structured interview that assesses multiple features of suicidality by asking about historical suicidal behavior and intent, as well as past-month suicidal behavior and intent. The C-SSRS has demonstrated good internal consistency and convergent validity [47].

#### Primary outcome measure

*Overall Anxiety Severity and Impairment Scale (OASIS)*. The OASIS [48] will be administered as a transdiagnostic measure to assess past-week severity, frequency, and impairment due to symptoms of any anxiety disorder. The OASIS is a five-item self-report measure, with items rated on a 5-point scale from 0 (little or none) to 4 (extreme). A cut-score of 8 is used to determine anxiety levels consistent with an anxiety disorder diagnosis [49]. Good convergent validity and internal consistency have been demonstrated for OASIS in previous studies [8].

#### Secondary outcome measures

Secondary outcome measures are outlined in Table 3. These include measures of each DSM-V-TR anxiety disorder, measures of co-occurring depressive symptoms, and minority stress measures.

**Table 3:** Secondary outcome measures.

Outcome measure	Construct variable and validity	Number of items	Internal consistency (*α*)	Type of validity (*r*)
Overall Depression Severity and Impairment Scale (ODSIS) [50]	Severity and impairment of past-week depression symptoms	5	.93 [51]	Convergent, .67 [51]
Generalized Anxiety Disorder Dimensional Scale (GAD-D) [52]	Generalized anxiety symptom severity	10	.94 [53]	Convergent, .77 [53]
Social Anxiety Disorder Dimensional Scale (SAD-D) [52]	Social anxiety symptom severity	10	.94 [54]	Convergent, .74 [54]
Panic Disorder Dimensional Scale (PD-D) [52]	Panic disorder symptom severity	10	.94 [52]	Predictive, .58 [52]
Agoraphobia Dimensional Scale (AG-D) [52]	Agoraphobia symptom severity	10	.98 [52]	Predictive, .82 [52]
Specific Phobia Dimensional Scale (SP-D) [52]	Specific phobia symptom severity	10	.96 [52]	Predictive, .70 [52]
Separation Anxiety Disorder Dimensional Scale (SepAD-D) [55]	Adult separation anxiety disorder symptom severity	10	.90–.94 [56]	Convergent, .43–.53 [56]
Patient Health Questionnaire 9-Item (PHQ-9) [57]	Depressive symptom severity	9	.87 [58]	Convergent, .80 [58]
Lesbian, Gay, and Bisexual Identity Scale–Internalized Homonegativity Subscale (LGBIS-IHS) [59]	Internalized homophobia severity	3	.87 [59]	Convergent, .83–.85 [59]
Sexual Orientation Concealment Scale (SOCS) [60]	Sexual orientation concealment	6	.77 [60]	Convergent, .54 [60]
Rejection Sensitivity Questionnaire—Adult (RSQ-A) [61] ^a^	Rejection sensitivity	9	.70 [61]	Convergent, .52 [61]
Heterosexist Harassment, Rejection, and Discrimination Scale (HHRDS) [62]	Interpersonal stigma based on sexual orientation or gender identity	6	.90 [62]	Convergent, .35 [62]
NIMH Clinician Global Impression–Improvement (CGI-I) Scale [63] ^b^	Participant-rated and clinician-assessed global change in anxiety disorder symptom severity	1	N/A	Convergent, .71–.74 [64]
NIMH Clinician Global Impression–Severity (CGI-S) Scale [63] ^b^	Participants’ self-rated and clinician-assessed anxiety disorder symptom severity	1	N/A	Convergent, .59–.83 [64]

*Note*.

^a^ Minor adaptions were made in this study to the pronouns in each question by changing ’he/she’ to ’he/she/they’ due to the issue of binary gender wording in psychological research [65].

^b^ The CGI-S and CGI-I will be delivered in both self-report and clinician-administered formats.

#### Process/Acceptability measures

*Client Satisfaction Questionnaire (CSQ)* [66]. The CSQ is an 8-item measure of the participant’s satisfaction with the treatment they were provided. The scale has demonstrated sound internal consistency and convergent validity [66, 67].

*Working Alliance Inventory–Short Form Revised (WAI-SF)* [68]. The WAI-SF is a revised 12-item form of the Working Alliance Inventory [69] that measures three main aspects of therapeutic alliance: agreement on the goals of therapy, agreement on the tasks of therapy that will achieve the goals, and the development of an emotional bond. In previous research, the WAI-SR has demonstrated good internal consistency and convergent validity [70].

*Credibility/Expectancy Questionnaire (CEQ)*. The CEQ is a six-item questionnaire measuring a client’s expectancy of the treatment and the credibility of the treatment rationale. The questionnaire is widely used and has demonstrated good internal consistency and predictive validity in previous research [71].

*Acceptability Questionnaire (AcQ)*. The AcQ is a 10-item measure of the acceptability of treatment and includes open-text questions to collect feedback. The questionnaire has been used in other remote treatment trials [46].

*Service Use Questionnaire (SUQ)*. The SUQ is a two-item measure of any health professionals seen and treatments used by participants due to their anxiety symptoms since their first and last appointment. This is administered to understand confounding variables in treatment efficacy and treatment acceptability.

*Adherence Questionnaire (AdQ)*. The AQ is a single-item measure of time spent per day working on the skills described in treatment. Participants are asked to indicate how many minutes were spent per day using the skills presented in treatment.

#### Qualitative interviews

Eight to ten participants from both the immediate treatment and waitlist control group will be selected to participate in qualitative interviews to provide participant perspectives on treatment acceptability. The first 16–20 participants who enroll will be selected on the basis of a representative matrix that aims to achieve a maximally diverse sample, as per previous studies [72].

Interview topics were derived from a review of previous literature on evaluating acceptability in clinical trials [73] and from LGBTQ+ adapted CBT specific trials [23]. Interview topics revolve around treatment acceptability, and include motivation for enrolment, videoconferencing acceptability, treatment length, protocol content, perceived critical elements, mechanism of change, therapeutic alliance and modifications required. Interview audio will be recorded and transcribed. Strategies to maximize the trustworthiness of the research [74] will be employed (e.g., member checking, presentation of data verbatim, reflexive diarising and discussion). The design and reporting of the study will be guided by the Consolidated Criteria for Reporting Qualitative Research (COREQ) [75]. Wherever possible, a member of the research team who did not conduct the participant’s treatment will conduct the interviews.

#### Risk management

Risk assessments with associated referrals to crisis support services will be conducted at screening, assessment, and throughout treatment. Participants will be provided with mental health crisis resources through multiple communication modes, and an individualized safety plan will be developed for all participants in session one. Participants whose symptoms reliably deteriorate [76] or remain consistently elevated will be contacted to encourage contact with other healthcare providers. Participants in the control group are not prohibited from accessing other services and are encouraged to contact their primary care team and crisis services as needed. All significant safety issues or serious adverse events, while unlikely and unanticipated, will be recorded. The PI (BW) will notify the University of Technology Sydney Health and Medical Research Ethics Committee in the event of serious adverse events. As the intervention is non-invasive, low-risk, and non-pharmacological, a data monitoring committee will not be necessary for this trial. Instead, the PI and research team will conduct data monitoring, and will meet in fortnightly to monthly meetings with regular reviews of protocol adherence and trial progress.

#### Treatment

Table 4 outlines the content of each treatment session. The transdiagnostic CBT protocol is based on the *Unified Protocol for the Transdiagnostic Treatment of Emotional Disorders* treatment manual [77]. The treatment is transdiagnostic as it focuses on difficulties with anxiety and emotional dysregulation, common to many internalizing disorders. The goal of the Unified Protocol is to increase acceptance of strong emotions and reduce secondary judgments around these emotions so that participants can reduce unhelpful avoidance and meet their goals by responding to emotions in helpful ways [77]. The Unified Protocol’s efficacy for anxiety symptoms in eight-session protocols has been indicated in previous trials [78].

**Table 4:** Session-by-session protocol.

Transdiagnostic CBT based on the UP [77]		LGBTQ+ adapted transdiagnostic CBT based on the LGBTQ+ adapted UP [26]	
Session	Module	Session	Module
1	Psychoeducation on the CBT model	1	Psychoeducation on the CBT model
			Treatment goals
	Treatment goals		Understanding the nature and impact of LGBTQ+ related LGBTQ+ related stress ^a^
2	Mindfulness of emotions	2	Mindfulness of emotions
3	Building cognitive flexibility with restructuring	3	Building cognitive flexibility with restructuring
4	Addressing emotional avoidance and emotion-driven behavior	4	Addressing emotional avoidance and emotion-driven behavior
5	Behavioral exposures/experiments	5	Psychoeducation around assertiveness
			Behavioral exposures/experiments with a focus on assertiveness
6	Behavioral exposures/experiments	6	Behavioral exposures/experiments
7	Behavioral exposures/experiments	7	Behavioral exposures/experiments
8	Relapse prevention	8	Relapse prevention

*Note*.

^a^ Psychoeducation on the nature and impact of LGBTQ+ related stress is emphasized in session one, but is ongoing throughout each module and skill used in the protocol.

The LGBTQ+ adapted transdiagnostic CBT protocol is based on the *Transdiagnostic LGBTQ-Affirmative Cognitive-Behavioral Therapy* treatment manual [26]. The treatment will consist of the essential elements of the immediate treatment group’s intervention but includes adaptions based on minority stress theory [6] and qualitative needs-based interviews of the LGBTQ+ community [27]. The adaptation considers how normal, non-pathological coping strategies or responses to minority stressors might maintain anxiety or depressive disorder symptoms [5, 6]. It does this by prioritizing the installation of skills to cope with minority stress, such as new therapeutic techniques such as assertiveness experiments/exposures and minority stress-informed psychoeducation in each session. This allows for insight-building around the nature and impact of LGBTQ+ related stress in the LGBTQ+ adapted protocol that is not provided in the standard protocol. For example, the potential minority-stress based origins of thinking traps or unhelpful emotional behaviours are explored with each participant in the LGBTQ+ adapted protocol. An additional emphasis beyond what is explored in the standard protocol around encouraging participation in the LGBTQ+ community as an avenue to buffer LGBTQ+ related stress is also added in behavioural experiments, exposures, and relapse prevention.

The adaptation also aims to target minority stress-based thoughts and core beliefs where possible with the clinician drawing on the minority stress framework to select appropriate cognitions. For example, psychoeducation is provided on LGBTQ+ proximal minority stressors such as rejection sensitivity, concealment of identity, and internalized LGBTQ+ stigma, and the participant is invited to complete restructuring on beliefs related to these experiences. Additionally, for each skill provided in the original UP, the LGBTQ+ adapted UP provides examples or case vignettes that are minority stress-based to emphasize that the skills learned in cognitive behavior therapy can be applied and used to deal with minority stress.

Per previous trials [9, 24], clients will be assigned between-session therapeutic activities to complete between sessions. These activities may involve completing thought monitoring forms, doing mindfulness exercises, completing behavioral experiments or exposures, and so on. Time spent on these activities is unlikely to exceed three hours per week and will vary week-to-week based on the schedule and individual participant goals.

To feasibly deliver both protocols in an Australian psychology community-based context [79], treatment will be delivered individually over eight 50–60-minute weekly sessions. All sessions will be manualized and delivered via the videoconferencing software Zoom. Zoom was selected due to its data security measures and compliance with the HIPAA requirements for healthcare [80]. Clinicians delivering videoconferencing treatment will be based in secure University of Technology Sydney facilities. Videoconferencing-delivered treatment has demonstrated equivalence to standard treatment in previous studies [81].

Sessions will be audio recorded to ensure treatment fidelity by having the PI (BW) review and score fidelity for 10% of sessions using standardized session checklists. Registered or provisionally registered psychologists in their final year of the UTS Master of Clinical Psychology will deliver treatment, and all clinicians will be under the supervision of an experienced clinical psychologist (BW). All clinicians will be trained thoroughly by the project investigators in the administration of the treatment protocol.

### Analysis

#### Data storage

To ensure confidentiality, all trial data will be securely stored in accordance with data safety standards and the guidelines of the National Health and Medical Research Council [82]. Trial data will be de-identified prior to analysis and will only be available to researchers. All trial data will be stored on a restricted access and secure network drive for fifteen years [82]. De-identified results are expected to be communicated to the public via publication, conference presentation, or professional media communications.

#### Statistical analysis

For the quantitative data, mixed linear models analysis will be used to understand the efficacy of the treatment on participant primary and secondary outcomes. The general linear mixed model is a robust analysis that can be used for longitudinal and repeated measure clinical trial data [83]. These analyses will use maximum likelihood estimation and an appropriate covariance structure to provide unbiased estimates in the event of missing data under the assumption of data missing at random [83]. Conservative intent-to-treat principles will be used to handle missing data. Within-group and between-group effect sizes (Cohen’s *d*) will also be calculated.

Secondary analyses may include intersectional moderator analyses of treatment efficacy, as appropriate, based on the person-level stigma moderators and distal minority stressors in the minority stress model [6]. Comparisons between previous research into the standard treatment and the LGBTQ+ adapted CBT protocol will be compared the current results using benchmarking analyses [84].

Clinically significant change will be assessed in three ways. The first will be the change in diagnosis, as assessed by the proportion of participants who no longer meet diagnostic criteria for their primary disorder as assessed by the DIAMOND [45]. Secondly, clinically significant change will be assessed by the proportion of participants whose OASIS score changes from above the clinical cut-score of eight [48] to below the cut-score at post-treatment and three-month follow-up. Thirdly, treatment response will be defined using the reliable change index (RCI) of Jacobson and Truax [76] to compute reliable and clinically significant change for participant symptoms accounting for measurement error, as per previous trials [85].

To qualitatively understand treatment acceptability, a framework analysis will be conducted using interview data with NVivo12 [86]. First, interviews will be transcribed, read, and re-read to familiarise researchers with the interview content. Then, transcripts will be coded to capture the meaning of participant’s perspectives while condensing the interview content. Codes will be cross-checked with the research team to ensure rigour and data trustworthiness. Codes will then be collapsed and sorted into themes using a thematic map to provide an overview of the data. Then, themes will be refined for coherence before being finally decided and named.

The data will be collected, analysed, and synthesised from a critical realist perspective, which supposes that the perspectives articulated by participants are understood and interpreted through the filter of human senses and experiences [87]. As such, critical realism is well suited to analysis of social issues and to suggesting solutions to social problems [87]. Selected relevant quotes will be reported to contextualize the quantitative findings. To provide thick data to allow researchers to appraise the transferability of participant perspectives, qualitative themes will be reported separately where possible. Themes will be reported separately but in reference to quantitative findings to contextualize the interpretation of treatment efficacy results and suggest improvements in future trials and CBT implementation.

The model of trustworthiness proposed by Lincoln and Guba [74] will be followed to ensure qualitative methodological rigor and mitigate potential biases in qualitative research. This involves embedding credibility, transferability, dependability, and confirmability throughout the research process. The following strategies recommended by Nowell and colleagues [88] will be followed to embed these processes in the research; prolonged data engagement; reflexive journaling (i.e., memos); well-organized storage of data, field notes, memos and related documents; debriefing with peers; for referential adequacy of candidate themes, returning to raw data, and; providing detailed description of the research context and an audit trail. Lincoln & Guba’s recommendations around transferability [74] through providing thick descriptions of participant perspectives will aid in mitigating interviewer bias, along with a regular reflexive journalling and debriefing around the administration of a semi-structured interview guide by a separate clinician to the participant’s therapist.

#### Power

GLIMMPSE 2.0.0 [89] was used to calculate the approximate sample size required to determine within-and between-group differences using mixed linear models with an alpha of .05 and power of .80 per convention in social sciences. The model for estimated power was based on a previous controlled trial of the Unified Protocol for people with anxiety disorders across three time-points compared to waitlist control [90]. To detect within-group effects across three time-points in the LGBTQ+ adapted arm, the means from a previous trial of the LGBTQ+ adapted Unified Protocol [8] were used. The psychometric properties of the OASIS were extracted from an evaluation among outpatients with diagnosed anxiety disorders [91]. The power analysis results indicated that a total sample size of 52, with 26 participants in each group, would be required to detect effects comparable to previous trials with power of .80, per convention in social sciences [92]. To detect within-group effects in the LGBTQ+ adapted arm, a total estimated sample size of 23 was required to detect effects comparable to previous trials.

Using anxiety disorder remission rates from meta-analytic research [93, 94], the power to detect clinically significant differences in diagnostic status was estimated to be sufficient with 42 participants in groups of 21 each [95]. In qualitative research, Braun and Clarke [96] recommend that 10 to 20 participants are typically sufficient to identify themes, with Malterud and Siersma [97] recommending that fewer participants may be needed provided the sample holds high information power. The qualitative sample size of 16–20 is consistent with other sample sizes in this area of research [8, 23].

## Conclusions

Anxiety disorders cause significant distress and impairment [2] and are more common among LGBTQ+ people than in the general population [1, 3]. While transdiagnostic CBT has early evidence of efficacy for LGBTQ+ people [8, 40], standard and adapted CBT has not yet been evaluated in individuals with primary anxiety disorders who identify as LGBTQ+. Therefore, the primary aim of this study is to examine the efficacy of standard CBT for LGBTQ+ individuals when compared with a waitlist control group. The secondary aims are to examine the efficacy of LGBTQ+ adapted transdiagnostic CBT in reducing symptoms of anxiety disorders in LGBTQ+ people with a primary anxiety disorder and to examine the acceptability and feasibility of the trial content and process. The results are anticipated to contribute to the evidence base around the efficacy of transdiagnostic, accessible, LGBTQ+ affirmative treatment for anxiety disorders in LGBTQ+ people and provide important insights into the acceptability and feasibility of these treatment protocols. Future clinical trials using larger samples and active control groups will be required to further examine the efficacy of the standard UP and adapted UP for LGBTQ+ people. Future research will also be needed to directly compare the efficacy of the two treatments, as well as examine the efficacy of each treatment amongst subgroups within the LGBTQ+ population.

## Trial status

Version 2.3, 7 May 2024. Important protocol modifications, such as changes to eligibility criteria, outcomes, or analyses, will have approval sought from the UTS MREC and will be updated in the clinical trial registry for trial accountability and transparency.

## Supporting information

S1 Table SPIRIT reporting checklist. (DOCX)

S2 Table TIDieR reporting checklist. (DOCX)

S1 File (DOCX)
