# Supplementary material for: Efficacy of videoconferencing-delivered cognitive behavioural therapy to reduce anxiety disorder severity in LGBTQ+ people: An exploratory trial protocol
Source: PLoS One. 2025 Jan 24;20(1):e0316857. doi: 10.1371/journal.pone.0316857 (PMC11760579; doi:10.1371/journal.pone.0316857)
Supplement: S1 File — (DOCX) [file pone.0316857.s003.docx]

**Remote Cognitive Behavioural Therapy for LGBTQ+ People with Anxiety Disorders: An Exploratory Trial**

**Principal Investigator:**

A/Prof Bethany Wootton

Address: Discipline of Clinical Psychology. Graduate School of Health. PO Box 123. Broadway. NSW 2007.

Telephone: (02) 9514 3942/ 0428690393

Email: [bethany.wootton@uts.edu.au](mailto:bethany.wootton@uts.edu.au)

**Co-Supervisor:**

A/Prof Emma Power

Address: Discipline of Speech Pathology. Graduate School of Health. PO Box 123.

Telephone: [+61 (02) 9514 7348](tel:+61295147348)

Email: emma.power@uts.edu.au

**Co-Supervisor:**

Dr Liam Casey

Address: 622 Lygon Street, Carlton North VIC 3054

Telephone: (03) 7018 1718

Email: liam@drliamcasey.com

**Student (PhD) Investigator:**

Isaac Dunn (Student number: 14159180)

Address: Discipline of Clinical Psychology. Graduate School of Health. PO Box 123. Broadway. NSW 2007.

Telephone: 0481245650

Email: Isaac.Dunn@student.uts.edu.au

**Student (Master of Clinical Psychology) Clinicians:**

Nicola White (Student number: 24519961)

Address: Discipline of Clinical Psychology. Graduate School of Health. PO Box 123. Broadway. NSW 2007.

Email: [Nicola.F.White@student.uts.edu.au](mailto:Nicola.F.White@student.uts.edu.au)

Amy Wang (Student number: 24931038)

Address: Discipline of Clinical Psychology. Graduate School of Health. PO Box 123. Broadway. NSW 2007.

Email: Amy.Wang-3@student.uts.edu.au

Julia Goodman (Student number: 24931949)

Address: Discipline of Clinical Psychology. Graduate School of Health. PO Box 123. Broadway. NSW 2007.

Email: [Julia.Goodman@student.uts.edu.au](mailto:Julia.Goodman@student.uts.edu.au)

Maral Melkonian

Address: Discipline of Clinical Psychology. Graduate School of Health. PO Box 123. Broadway. NSW 2007.

Email: [maral.melkonian@student.uts.edu.au](mailto:maral.melkonian@student.uts.edu.au)

**Protocol Version** # 2.3

**Protocol Date:** 06.06.24

**Summary**

| **Overall project title:** | Remote Cognitive Behavioural Therapy for LGBTQ+ People with Anxiety Disorders: An Exploratory Trial |
| --- | --- |
| **Protocol version** | 2.3 |
| **Objectives** | **Primary**  The primary objective of this program of research is to understand how efficacious cognitive behavioural therapy (CBT) is when delivered via videoconferencing for anxiety disorders in LGBTQ+ adults.  **Secondary**  The secondary objectives of this project are: 1) to benchmark the efficacy of adapted CBT compared to standard CBT for LGBTQ+ adults, when both are delivered via video-conferencing; 2) to understand the feasibility of delivering CBT to LGBTQ+ adults via video-conferencing; and 3) understand the perspectives of LGBTQ+ participants on the content, delivery format, and acceptability of CBT delivered via telehealth for anxiety disorders. |
| **Study design** | A randomized controlled trial (RCT) compliant with CONSORT-R guidelines, comparing an immediate treatment group with a waitlist control group, with a nested qualitative study to examine acceptability of the treatment. |
| **Planned sample size** | A total sample of 52 participants, with 26 participants in each group. Qualitative interviews will use 8 - 10 participants from each intervention group, with a total of 16 - 20 included participants in qualitative evaluation interviews. |
| **Selection criteria** | RCT Inclusion criteria: (1) Residing in Australia currently; (2) aged 18 years or over; (3) Fluent in English; (4) Meets criteria for an anxiety disorder as the primary diagnosis and the disorder is of at least ‘moderate severity’ (defined as a score of 4 on the DIAMOND module severity measure); (5) On a stable dose of psychotropic medication or medication free; (6) Not receiving regular psychological services currently for their anxiety symptoms (defined as sessions at least once a week with a qualified mental health professional); (7) Identify as LGBTQ+, and; (8) Has access to a private location with internet to complete the treatment for the duration of the study.  RCT Exclusion criteria: (1) Suicide risk as assessed by item 9 of the PHQ-9 (a score of 2 or above) at baseline, a score of ‘moderate’ or higher on the C-SSRS, responses of ‘yes’ on item 1-3 on the risk questionnaire, or via clinician judgement during the diagnostic interview; (2) Daily use of alcohol or illicit drugs (meaning illegal drugs, pharmaceutical drugs used outside of their prescribed or intended use, and other substances inappropriately used); (3) The presence of a schizophrenia spectrum disorder as assessed by the DIAMOND semi-structured clinical interview; (4) Any significant cognitive/intellectual impairment as assessed during the diagnostic interview; (5) A medical condition that may interfere with treatment; (6) No access to a computer with a camera and stable internet on a regular basis; (7) Is not willing to engage in treatment on a regular basis using internet-videoconferencing software; (8) Does not identify as non-heterosexual or non-cisgendered; (9) Does not indicate any anxiety disorder symptoms on the DIAMOND Self Report screener. |
| **Study procedures** | Recruitment will use a variety of methods (outlined below), all of which will include a link for participants to access and consent to the Participant Information and Consent Form (PICF). After consenting to the PICF, participants will proceed through the study in the procedure outlined below:   1. Complete online screening questionnaires, including baseline questionnaires to assess for key inclusion/exclusion criteria. 2. Complete videoconferencing-delivered diagnostic interview to assess diagnostic status and remaining inclusion/exclusion criteria. 3. Eligible participants will be randomised to either immediate treatment or waitlist control (randomisation procedures described below). 4. Participants in the immediate intervention group will receive 8 sessions of videoconferencing-delivered, non-adapted cognitive behaviour therapy as per the Unified Protocol manual (described below). 5. Participants randomised to the waitlist control group will receive treatment after a waitlist period of 8 weeks. Participants in the waitlist control group will receive 8 sessions of LGBTQ-Affirmative CBT delivered via videoconferencing (described below). 6. Participants will complete four sets of self-report measures, at baseline, mid-treatment, post-treatment, and 3-month follow up. 7. Participants’ diagnostic status will be assessed with the DIAMOND semi-structured interview at pre-treatment, post-treatment and 3-months follow up. 8. Participants will be invited to attend a 30 to 60-minute qualitative semi-structured interview after their post-treatment interview. |
| **Statistical considerations** | **Sample size calculation**  The sample size calculator developed by Faul et al. (2007) was used to calculate a total sample size of 52 participants, in samples of 26 for each group, to detect a large effect (*d* = .80) with 80% power. Previous research has indicated that CBT has a large effect size for anxiety disorders (Cuijpers et al., 2013). To detect clinically significant differences in participant diagnostic status, 42 participants will need to be recruited, in samples of 21 in each group, using the ClinCalc LLC (2024) sample size calculation tool.  A total of 16 – 20 participants will be selected for inclusion to the qualitative study, with eight to ten participants each from each intervention group. Braun and Clarke (2013) recommend that 10 to 20 participants are typically sufficient to identify themes, although, previous researchers have suggested that fewer participants may be needed if the sample holds more information power. This sample size is consistent with other sample sizes in this area of research (Jackson et al., 2022; Pachankis et al., 2020). Participants will be selected based on a representative matrix to achieve a maximally diverse qualitative sample, consistent with other qualitative studies (Christensen et al., 2023).  **Analysis plan**  To ensure analyses manage missing data conservatively, intent-to-treat principles and mixed-linear models will be used for quantitative analyses. Mixed-models are used to analyse longitudinal clinical trial data robustly. Using an appropriate covariance structure and maximum likelihood estimation will provide unbiased estimates in the event of missing data, assuming that data is missing at random. Qualitative data will be analysed using framework analysis (Ritchie & Spencer, 1994). |
| **Study duration** | 5 years |

**BACKGROUND**

Internationally, approximately 18 - 37% of adults will develop an anxiety, mood, or related disorder in their lifetime (McGrath et al., 2023). These disorders are known as internalising disorders because of their tendency to turn inwards in response to stressors, which results in high levels of anxiety, distress, and negative affect (Krueger, 1999). These are differentiated from externalising disorders, which have a tendency to respond to with stressors with outward coping mechanisms such as excessive use of alcohol and other substances. Depressive disorders can include conditions such as major depressive disorder and persistent depressive disorder, and anxiety disorders often include conditions such as generalised anxiety disorder, panic disorder, and social anxiety disorder. These disorders have significant impacts on people’s functioning (Vigo et al., 2016) and quality of life (Hohls et al., 2021), and represent a large burden on people’s lives (Greenberg et al., 2021; Konnopka & König, 2020).

In Australia, while the general population’s 12-month prevalence rate of internalising disorders is high at 8 – 17%, it is two to three times higher among lesbian, gay, bisexual, and other non-heterosexual people at approximately 28% to 50% (Australian Bureau of Statistics, 2023). Similarly, approximately one in three gender-diverse people were reported to have experienced an internalising disorder in the past 12 months (Australian Bureau of Statistics, 2023). This higher rate of internalising disorders in LGBTQ+ people is not due to any inherent psychopathology: rather, the higher rate of stigma and discrimination experienced by these groups is proposed to account for these disparities (Hatzenbuehler, 2009; Meyer, 2003). The excess stress of dealing with chronically high levels of stigma and discrimination is called minority stress (Meyer, 2003). While structural and social-level change is needed to reduce minority stress in LGBTQ+ people, implementing this change can take significant time (Hinshaw & Stier, 2008) and may be incomplete in addressing the issue effectively (Kalev et al., 2006). Thus, tailored treatments for LGBTQ+ unique minority stressors and common internalising disorders are needed to support a reduction in symptoms and quality of life in LGBTQ+ people (Barrera & Castro, 2006).

Cognitive behaviour therapy (CBT) is recommended as the gold-standard, first-line treatment for internalising disorders (American Psychological Association Presidential Task Force on Evidence-Based Practice, 2006; Australian Psychological Society, 2018; National Institute for Health and Care Excellence, 2011). CBT’s effectiveness has been shown across 106 meta-analyses, with a medium to large effect size for treating any internalising disorder (Hofmann et al., 2012). Comorbidity between internalising disorders is high (Kessler et al., 2011), and treatments have been developed to address multiple internalising disorders simultaneously. This is known as transdiagnostic treatment. The Unified Protocol (Ellard et al., 2010) is a well-researched type of transdiagnostic treatment that has been demonstrated to have large effect sizes for internalising disorders in the general population (Sakiris & Berle, 2019). Additionally, preliminary controlled trials have shown that an LGBTQ-adapted Unified Protocol for LGBTQ+ adults is also effective at reducing symptoms of internalising disorders (Ellard et al., 2010; Pachankis et al., 2015; Pachankis et al., 2020b). In two randomised control trials of LGBTQ+ adapted CBT, Pachankis et al. (2015) and Pachankis et al. (2020) found comparable effect sizes pre- to post-treatment for anxiety and related disorders and depressive symptoms as in trials of the Unified Protocol in the general population. It is important to understand if LGBTQ-adapted CBT is more effective than standard CBT, but to date, no clinical trials have directly compared the two treatments or measured standard CBT’s efficacy in LGBTQ+ people. This study will contribute to the emerging evidence of equivalence between the two treatments by understanding the preliminary efficacy of both interventions, and assessing feasibility to progress to a larger trial where both treatments are directly compared.

Similarly, few studies have used telehealth-delivered CBT for internalising disorders in LGBTQ+ adults (Van Der-Pol Harney & McAloon, 2018). Despite this, remotely delivered CBT has demonstrated effectiveness for internalising disorders (Matsumoto et al., 2021). LGBTQ+ people face logistical barriers that may prevent them from seeking care, including exclusion from healthcare from non-affirming/non-educated providers or a desire to conceal their ‘outness’ by accessing affirming care (Foy et al., 2019; Olfson et al., 2000). Remotely delivered CBT provides a solution to mitigate these barriers for LGBTQ+ people but is yet to be explored in the literature.

**OBJECTIVES, DESIGN, AND HYPOTHESES**

The purpose of this study is to examine the efficacy, feasibility, and acceptability of videoconferencing-delivered CBT for anxiety disorders in LGBTQ+ adults. A CONSORT-R compliant, two-group, randomised control feasibility trial (RCT) will examine the research questions. The hypotheses for this study are below:

1. Videoconferencing-delivered, standard CBT will result in significant reductions in symptoms with a large between-group and within-group effect size at post-treatment and three-month follow-up.
2. Videoconferencing-delivered, LGBTQ-adapted CBT will result in a similar degree of symptom reduction when compared to standard CBT.
3. Videoconferencing-delivered standard CBT and LGBTQ-adapted CBT will be feasible to deliver to LGBTQ+ adults with diagnosed anxiety, as assessed by at least 80% treatment fidelity (Borrelli et al., 2005), at least 80% retention following the waitlist period (Fernandez et al., 2015).
4. Videoconferencing-delivered CBT will be acceptable to LGBTQ+ adults with diagnosed anxiety disorders, as assessed in qualitative semi-structured exit interviews and by at least 70% treatment retention and questionnaire completion at follow-up (Fernandez et al., 2015).

The study’s design has two groups:

**Group 1 (*n* = 26): Immediate treatment group.** Group 1 will immediately receive standard, unadapted CBT delivered via videoconferencing. This treatment will be delivered via Zoom. Participants in Group 1 will receive one 50-minute session per week for eight weeks.

**Group 2 (*n* = 26) Waitlist control group.** Group 2 will commence the treatment after a waitlist period of nine weeks, and will recomplete screening measures and their diagnostic interview. Group 2 will receive a similar CBT treatment delivered via Zoom, but this treatment has been tailored to account for the unique challenges experienced by LGBTQ+ people (Pachankis, 2014). LGBTQ-adapted treatment has been shown to be efficacious in previous trials (Pachankis et al., 2015; Pachankis et al., 2020).

A waitlist control was chosen given no studies have currently investigated standard CBT for internalising disorders in LGBTQ+ individuals. As such this study is designed as a feasibility study, phase 1 clinical trial.

**PARTICIPANT NUMBERS & POWER**

Using G*Power, it was determined that 52 participants would be needed to detect a large, post-treatment, between-group effect size with alpha set to 0.05 and 80% power (Faul et al., 2007). This is because previous meta-analyses have detected large effect sizes for the effect of CBT on anxiety (Cuijpers et al., 2013). Previous preliminary randomised control trials of CBT for LGBTQ+ adults with both internalising and externalising disorders have recruited samples of similar sizes (Pachankis et al., 2015; Pachankis et al., 2020).

Power was also calculated to detect clinically meaningful changes in participant anxiety disorder diagnosis according to DSM criteria. Using the ClinCalc LLC (2024) sample size calculation tool, the recruitment of 42 participants would be sufficient to detect a post-treatment between-group clinically meaningful change in anxiety disorder diagnosis with 80% power and alpha set to 0.05. This is based on the anticipated incidence of anxiety remission drawn from the metanalysis of Springer et al. (2018), which found that 55.8% of anxiety disorder diagnoses remitted after receiving CBT. For waitlist controls, anxiety disorder symptoms in participants in waitlist control fell below the clinical cut-score 15.4% of the time (Scott et al., 2022). Given CBT has not been examined in trials of LGBTQ+ participants with only internalising disorders (Van Der Pol-Harney & McAloon, 2018), an exact power analysis is unable to be determined and is thus an exploratory estimate. However, 95% confidence intervals will be reported with any effect estimates to determine the intervention's precision.

A total of 16 – 20 participants will be selected for inclusion in the qualitative study, with eight to ten participants each from groups one and two (after receiving treatment). Braun and Clarke (2013) recommend that 10 to 20 participants are typically sufficient to identify themes, although, suggested that fewer participants may be needed if the sample holds more information power (Malterud et al., 2016). This is because four of the five aspects of the information power model suggested by Malterud et al. (2016) indicate higher information power and, thus, a smaller sample size in this study. This is indicated by the narrow aim of the qualitative study (to understand the feasibility and acceptability of telehealth-adapted CBT in LGBTQ+ participants), the specificity of the population (LGBTQ+ participants with diagnosed depressive or anxiety disorders who have received remotely-delivered CBT), the use of CBT and minority stress theory, and strong dialogue potential given the interviewer will have researched the subject area and will be using an interview guide. This sample size is consistent with other sample sizes in this area of research (Jackson et al., 2022; Pachankis et al., 2020). Participants will be selected based on a representative matrix to achieve a maximally diverse qualitative sample, consistent with other qualitative studies (Christensen et al., 2023). A lack of characterisation of diverse groups has been an issue in previous studies (Hall et al., 2019)

**PARTICIPANT RECRUITMENT**

Recruitment of participants will use a variety of methods. Flyers will be placed across University of Technology Sydney campus and other community noticeboards (Appendix A), on social media platforms like Facebook, LinkedIn, and Instagram (Appendix B), on Twitter (Appendix C), on professional networking sites for psychologists (Appendix D) and sent via email or letter to health professionals in Australia such as general practitioners, psychologists, counsellors, social workers, and psychiatrists (Appendix E). The student investigator may also directly approach health professionals and health services by phone, email, or in person to inform them of the study and provide the approved recruitment flyer. Health professionals or services that serve LGBTQ+ clients will be prioritised for contact. Social media posts will be made on the research team’s professional social media accounts. The moderators of relevant social media sites will be approached to advise of the potential for clients to access support through the study. Agreement will be obtained from at least one moderator before posting any approved script. Paid Google Advertisements may be posted (Appendix F) periodically. Applications to relevant not-for-profit organisations (Appendix G) will be made to advertise the study via their website and social media pages, including One Door Mental Health (<https://www.onedoor.org.au/resources/referral-forms/external-research-study-application-form>) and SANE Australia (<https://www.sane.org/adrc/external-research-projects>).

Participants for the qualitative nested study will be selected from the treatment sample using a representative matrix. This will ensure a broad representation of different participants perspectives and will be based on scores of the post-treatment Client Satisfaction Questionnaire (CSQ), sexual orientations, gender identities, racial and ethnic diversity, geographically located participants, and degree of completion of treatment.

All participants will be advised that as part of the study, they may be invited to a qualitative interview to understand their experience and preferences during the treatment. This will be included in the initial PICF form, and participants will be given the option to change their mind or decline participation in the qualitative interview with no impact on their relationship with their assigned clinician or the research team. Participants who completed at least one treatment session would be invited to attend the qualitative interview after completing their second structured diagnostic interview. An email will be sent to all participants selected to attend prior to their second diagnostic interview, which is attached in Appendix H. Should a participant be selected on the basis of the representative matrix, and decline, then the next participant with demographic characteristics in the representative matrix that has not yet included in the qualitative study will be selected to participate in the qualitative study.

**STUDY PROCEDURES**

Prior to engaging with the study, potential participants can read about it via the recruitment methods described above, or on the UTS-affiliated website for the study and related studies by participants (<https://www.uts.edu.au/about/graduate-school-health/clinical-psychology/what-we-do/clinical-psychology-research/telepsych-laboratory>).

All recruitment methods will include a link for participants to access and consent to the PICF. After consenting to the PICF, participants will proceed through the study in the procedure outlined below and in Figure 1:

**1. Online Screening**

Participants will follow a REDCAP link to read the online PICF. Participants who consent will then complete the demographic form, the DIAMOND screener, and the PHQ-9 to assess suitability. This is anticipated to take 20 – 30 minutes. Participants will provide their name and contact details (phone number and email address) for an interviewing clinician to contact them to complete the videoconferencing delivered diagnostic interview. Participants will be asked to indicate their preferred times for diagnostic screening.

Participants who do not meet the criteria for the study will be shown a screen providing information on how to access support and crisis services. Non-eligible participants will be informed if it is likely they meet the criteria for one of the disorders and will be encouraged to seek support from appropriate services such as their GP (Appendix I).

**2. Diagnostic Screening**

Based on the online screening questionnaires, participants who meet all the study criteria will be invited to a diagnostic interview using the DIAMOND (Appendix J). The purpose of the interview is to confirm their diagnostic status and assess comorbid conditions. Participants will be asked to confirm their email address and preferences for treatment on a particular day or time. Diagnostic interviews will be conducted via videoconferencing and will be audio-recorded. Once diagnostic criteria are confirmed, these participants will be randomised to a treatment condition. Diagnostic screening is anticipated to take 1 – 2 hours but may be longer for some participants.

If a participant does not meet the study criteria at this stage, they will be informed by one of the research staff via email (Appendix K) after the student clinician and chief investigator have confirmed they do not meet criteria via discussion. Then, an investigator will contact the participant to discuss treatment options. These participants will be encouraged to access support from their GP or appropriate services, including crisis services if necessary.

**3. Randomisation**

If a participant meets all the study criteria, then they will be randomised to either an immediate treatment group or a waitlist control group (WLC). Randomisation will be conducted using a random number generator via the website www.random.org to ensure unbiased selection. Assessing clinicians will not be aware of the randomisation status of participants and will be advised of their participants’ group randomisation by the Principal Investigator. Participants will be advised of their group assignment, along with their acceptance into the study, via email. An email with a REDCAP link to pre-treatment questionnaires will be sent to participants in both groups to be completed. After the competition of these questionnaires, participants in group 1 (immediate treatment) will be called to schedule treatment to begin as soon as possible. Participants in group 2 (waitlist control) will be called 9 weeks later to complete the diagnostic interview and self-report questionnaires and schedule treatment as soon as possible.

**4. Questionnaire Administration (pre-treatment, post-treatment, follow-up)**

All participants will complete the symptom screeners online via REDCAP before starting treatment (pre-treatment), after treatment completion (post-treatment), and three months after treatment (follow-up). These questionnaires will be automatically emailed via REDCAP at the allocated time. It is anticipated that it will take 20 minutes to complete the self-report questionnaires at each time point.

The diagnostic interview (DIAMOND) will be administered at post-treatment and follow-up via phone or videoconferencing (and before commencing treatment for the control group). Participants will be contacted to schedule this appointment, and ideally this appointment will be scheduled in the final treatment session. Only the modules that the participants met criteria for at pre-treatment will be administered.

**5. Qualitative Interview**

Eight to ten participants from both group one and group two (after commencing treatment), with a total of 16 – 20 participants, will be selected for inclusion to the qualitative interview, consistent with sample size in this area of the research (Jackson et al., 2022; Pachankis et al., 2020). Participants who consent to be interviewed in their initial PCIF will be selected based on a representative matrix that aims to achieve a maximally diverse sample, as per previous studies (Christensen et al., 2023). Wherever possible, a member of the research team who did not conduct the participant’s treatment will conduct the interviews. Masters students who conduct the interviews will be supervised by members of the research team.

The interview scheduled for a time that suits participants after the diagnostic interview and is expected to take approximately 30 to 60 minutes. For those who drop out or do not attend regular appointments, they will be still be contacted to organise an appointment time that suits them if they would like to participate. Field notes will be written immediately after each interview and organised as observational notes, theoretical notes, methodological notes, and analytical memos as suggested by Groenewald (2004). The interview topic guide is available in Appendix L, with support services to be provided to participants after the interview in Appendix M. The interview topic guide was derived from review of previous literature on evaluating acceptability in clinical trials (Sekhon et al., 2017), and from LGBTQ-adapted CBT specific trials (Jackson et al., 2022) and will be refined throughout the study as required as is best practice in qualitative research.

Interview audio will be recorded to assist with transcription. The benefits of the feedback on the intervention will be discussed at the beginning of the interview to encourage candid feedback in the semi-structured interview. Framework analysis (Ritchie & Spencer, 1994) will be used to analyse data under acceptability domains raised in the topic guide along with any additional inductive coding. Coding will be completed by the student investigator in a collaborative process with other investigators.

Design and reporting of the study will be guided by relevant guidelines, including the Consolidated Criteria for Reporting Qualitative Research (COREQ) (Tong et al., 2007). Strategies to maximize the trustworthiness of the research (Lincoln & Guba, 1985) will be employed (e.g., member checking, presentation of data verbatim, reflexive diarising and discussion).

**6. Managing Study Procedure Risks**

We expect participants will experience clinically significant improvements in the symptoms of their anxiety disorder. We also hope that participants will apply the strategies learnt in this trial after study completion, which will likely lead to long-term management of their symptoms. The protocol for treatment encourages participants to not just learn but practice techniques to manage their mental health conditions’ symptoms. It is important to note that in the short-term, participants in cognitive behavioural therapy can experience an increase in symptoms of a mental health condition; however, this is only a temporary increase and will diminish as treatment progresses (Reinecke et al., 2018).

At pre-treatment, post-treatment, and follow-up, participants will be given the option to choose whether their health care provider(s) are made aware of their participation in the study. The templates for these letters are available in Appendix N.

Supporting participant’s safety and emotional well-being is a priority of the researchers. In the rare circumstance that someone requires psychiatric consideration or experiences significant distress, the investigators are mental health clinicians who can assess the situation and refer the person to appropriate services such as their primary physician or emergency services. Given that this study is not trialling the effects of a pharmaceutical drug or medical equipment but rather the effects of talk therapy, potential adverse events are rare. In the unlikely event that adverse events do occur, a log will be kept of adverse events that arise. Serious adverse events (SAEs) involving an immediate physical risk to participant safety will be reported to the Principal Investigator, who will advise the Human Research Ethics Committee of the nature of the SAE and any actions taken to mitigate its effects.

It is noted by the researchers that most people presenting for treatment with an internalising disorder have experienced their symptoms for several years (Patton et al., 2014). This means that participants have likely consulted multiple medical or mental health clinicians without complete or partial remission of symptoms. Minority stressors for LGBTQ+ participants are also by nature managed by LGBTQ+ people chronically in the long-term (Meyer, 2003), and thus unlikely to need urgent care within the eight-week period of the waitlist, while receiving treatment for eight weeks, or during follow-up. In any case, this study is not a crisis service, and participants who need urgent care will be referred out at assessment to seek urgent psychiatric care or support from their primary care provider.

Management of risk will be ongoing throughout the project. Importantly, all participants will be made aware of what steps they can follow in a mental health emergency in multiple communication modes at multiple timepoints. For example, which services to contact and what to do in a mental health crisis is outlined in (1) the PICF; (2) a safety plan provided to all clients at the beginning of treatment; (3) in every email communication with participants. Secondly, participants' responses to their questionnaires will be monitored, meaning that deteriorating or consistently elevated symptoms will trigger the research team to contact the participant to encourage contact with their health care team or emergency services. All communication between participants and the research team will be documented.

Assessed for eligibility.

Participants who do not meet criteria will be excluded.

Three-month follow-up diagnostic assessment.

Post-treatment diagnostic assessment and self-report questionnaires.

Qualitative Interviews (n = 10)

Allocated to immediate intervention with Unified Protocol (n = 26).

Post-treatment diagnostic assessment and self-report questionnaires.

Qualitative Interviews (n = 10)

Three-month follow-up diagnostic assessment.

## Allocation

## Follow-up

## Post-treatment

Randomized (n = 52)

Analysed

Analysed

## Analysis

Treatment phase

**Figure 1.**

*Study Flow Diagram*

Pre-screened for eligibility.

Participants who do not meet criteria will be excluded.

Allocated to waitlist control condition with LGBTQ-Affirmative Unified Protocol after 9-week waitlist period ends (n = 26).

Diagnostic status and self-report measures re-administered prior to treatment.

**STUDY MEASURES**

***Online Screening Measures***

The questionnaires that participants will complete for eligibility screening in REDCAP are outlined below, and are shown in detail in Appendix P.

- **Demographic Form:** A basic demographic questionnaire will collect information on age, location, gender identity, sexual orientation, marital status, employment and education status, racial identity, access to a computer in a private space, and medication use.
- **DIAMOND Screener:** The DIAMOND screener (Tolin et al., 2018) is a 30-item self-report questionnaire which is used to indicate to the clinician which modules of the DIAMOND need to be administered during the semi-structured diagnostic interview. Only items endorsed on the DIAMOND screener are delivered during the DIAMOND interview.
- **Risk Questionnaire:** This risk questionnaire is a standard five-item questionnaire used to assess risky behaviours such as deliberate self-harm and problematic alcohol and/or illicit drug use. It has been used in online treatment studies (Wootton et al., 2019a) to screen for participants whose risk level may be too high to be seen in a remote treatment setting.
- **Patient Health Questionnaire 9-Item (PHQ-9) (Kroenke et al., 2001):** The PHQ-9 is a 9-item measure of depressive symptoms. Participants rate how frequently they experienced each symptom in the past two weeks on a four-point Likert scale from zero (*not at all*) to three (*nearly every day*). Scores of 10 or greater indicate clinically significant depression symptoms (Manea et al., 2012). The PHQ-9 has been demonstrated to have good psychometric properties in previous studies (Kroenke et al., 2001; Zuithoff et al., 2010).

***Diagnostic Screening***

Participants’ diagnostic status will be assessed using the *Diagnostic Interview for Anxiety, Mood, and Obsessive-Compulsive and Related Neuropsychiatric Disorders (DIAMOND)* (Tolin et al., 2018). The DIAMOND is a semi-structured diagnostic interview that assesses the DSM-5 diagnostic criteria for anxiety disorders, mood disorders, obsessive compulsive and related disorders, trauma and stressor related disorders, schizophrenia spectrum disorders, eating disorders, somatic symptom and related disorders, substance use disorders, and selected neurodevelopmental disorders. The DIAMOND has been shown to have acceptable reliability and validity for mood disorders and anxiety and related disorders (Tolin et al., 2018). Assessment modules will be administered if the DIAMOND screener indicates that the modules should be administered.

The five-item *Columbia Suicide Severity Rating Scale (C-SSRS)* (Posner et al., 2008) will be used to screen for risk of suicide over the past month in the diagnostic interview. Specifically, it assesses suicidal ideation and behaviour. The diagnostic screening will occur via videoconferencing and will be recorded.

***Outcome Measures***

***Primary measures***

The following disorder-specific measure will be completed by participants at pre-treatment, mid-treatment, post-treatment, and 3-month follow up.

- **Overall Anxiety Severity and Impairment Scale (OASIS; Norman et al., 2006):** The OASIS is a five-item self-report measure of the severity, frequency and impairment related to symptoms of anxiety in the past week. Items are rated on a 5-point scale from 0 (*little* or *none*) to 4 (*extreme*). A cut-score of 8 is used to determine anxiety levels consistent with an anxiety diagnosis. Good validity and internal consistency have been demonstrated for OASIS in previous studies (Campbell-Sills et al., 2009; Pachankis et al., 2015; Pachankis et al., 2020)

***Secondary Measures***

All participants will complete the following secondary self-report outcome measures at pre-treatment, mid-treatment, post-treatment, and three-month follow-up:

- **Overall Depression Severity and Impairment Scale (ODSIS; Bentley, 2014):** The ODSIS is a five-item self-report measure of the severity and impairment of past-week symptoms of depression from 0 (*little* or *none*) to 4 (*extreme*). A score of 5 or above has been used to determine clinical levels of depression (Mira et al., 2019). The ODSIS has been shown to have good validity and internal consistency in previous studies (Mira et al., 2019; Pachankis et al., 2015; Pachankis et al., 2020)
- **Generalised Anxiety Disorder Dimensional Scale (GAD-D) (LeBeau et al., 2012):** The GAD-D is a 10-item measure of generalized anxiety symptoms. Participants rate the past-month frequency with which they have experienced GAD symptoms on a five-point Likert scale ranging from 0 (*Never*) to 4 (*All of the time*). This results in a total score ranging between 0 and 40. The GAD-D has been shown to have acceptable psychometric properties (LeBeau et al. 2012).
- **Social Anxiety Disorder Dimensional Scale (SAD-D)** (Lebeau et al., 2012). The SAD-D is a 10-item self-report measure of social anxiety symptoms. Each item is rated on a five-point Likert scale ranging from zero (“*never*” or “*none*”) to four (“*all the time*” or “*extreme*”). The SAD-D has previously demonstrated good validity and internal consistency in previous samples (Lebeau et al., 2012).
- **Panic Disorder Dimensional Scale (PD-D)** (Lebeau et al., 2012). The PD-D is a 10-item measure of panic disorder symptoms. Items are rated on a five-point scale ranging from 0 to 4. The overall assessment is made by a total score, which is calculated by summing the scores for all ten items. The total scores range from 0 to 40. The PD-D has demonstrated good psychometric properties in previous studies (Lebeau et al., 2012).
- **Agoraphobia Dimensional Scale (AG-D)** (Lebeau et al., 2012). The AG-D is a 10-item scale with each item rated on a 5-point scale from 0 (*Never*) to 4 (*All of the time*). The total score can range from 0 to 40, with higher scores indicating greater severity of agoraphobia. The average total score is calculated by dividing the raw total score by 10, as this is the number of items in the measure.
- **Specific Phobia Dimensional Scale (SP-D)** (Lebeau et al., 2012). The SP-D is a 10-item measure that assesses the severity of specific phobia in individuals aged 18 and older. Each item asks the individual to rate the severity of specific phobia symptoms during the past 7 days. Each item on the measure is rated on a 5-point scale from 0 (*Never*) to 4 (*All of the time*). The total score can range from 0 to 40. Higher scores indicate greater severity of specific phobia.
- **Separation Anxiety Disorder Dimensional Scale (LeBeau et al., 2012):** The Separation Anxiety Dimensional Scale is a 10-item self-report questionnaire that examines the severity of separation anxiety symptoms in adults. Each item is rated on a five-point scale from 0 (*Never*) to 4 (*All of the time*). Total scores range from 0 - 40. Previous studies have demonstrated sound psychometric properties of the scale (Lebeau et al., 2012)
- **Patient Health Questionnaire 9-Item (PHQ-9) (Kroenke et al., 2001):** The PHQ-9 is a 9-item measure of depressive symptoms. Participants rate how frequently they experienced each symptom in the past two weeks on a four-point Likert scale from zero (*not at all*) to three (*nearly every day*). Scores of 10 or greater indicate clinically significant depression symptoms (Manea et al., 2012). The PHQ-9 has been demonstrated to have good psychometric properties in previous studies (Kroenke et al., 2001; Zuithoff et al., 2010).
- **The Lesbian, Gay, and Bisexual Identity Scale (LGBIS) (Mohr & Kendra, 2011).** The 3-item subscale of the LGBIS measures internalised stigma around one’s identity as a non-heterosexual person on a six-point Likert scale, where 1 is *strongly disagree* and 6 is *strongly agree.* The psychometric properties of the subscale have been demonstrated to be sound and have been used in previous clinical trials (Pachankis et al., 2023).
- **Sexual Orientation Concealment Scale (SOCS)** (Jackson & Mohr, 2016). The SOCS is a six-item measure of concealment of sexual identity. It uses a five-point Likert scale from 0 (*Not at all*) to 5 (All the time). The psychometric properties of the scale are sound with adequate reliability and validity (Jackson & Mohr, 2016).
- **Rejection Sensitivity Questionnaire - Adult (RSQ-A) (Berenson et al., 2009).** The RSQ-A is a 9-item scale that measures rejection sensitivity. Each item has one vignette which participants are asked to read and respond how concerned they would be about the situation and how likely it is they would make a positive attribution about the event. This is rated on a five-point Likert scale from how concerned the participant would be from 1 (*very unconcerned*) to 5 (*very concerned*) and how likely it is they would make a positive attribution, from 1 (*very unlikely*) to 5 (*very likely*). The questionnaire has demonstrated adequate psychometric properties in a prior sample (Berenson et al., 2009).
- **Heterosexist Harassment, Rejection, and Discrimination Scale (HHRDS) (Szymanski, 2006).** The HHRDS measures experiences in the past year of interpersonal stigma directed toward one’s sexual orientation or gender identity using a six-point Likert scale from 1 (*never*) to 6 (*almost all the time*). A total mean score is calculated, with higher scores indicating higher levees of stigma. Sound psychometric properties have been found in prior studies (Szymanski, 2006) and an adapted version of the questionnaire was used to be relevant to all LGBTQ+ people (Pachankis et al., 2023).
- **NIMH Clinician Global Impression (CGI) Scale (Guy, 1976)*:*** The CGI is a single-item measure of symptom severity and improvement. Severity scores range from 1 (*normal*) to 7 (*severely ill*) and improvement scores range from 1 (*very much improved*) to 7 (*very much worse*). The CGI-S and CGI-I will be delivered in both self-report and clinician-administered format.

The following measures will be administered to all participants at mid-treatment and post-treatment only:

- **Client Satisfaction Questionnaire (CSQ) (Larsen et al., 1979):** The CSQ is an 8-item measure of the participant’s satisfaction with the treatment they were provided. Higher scores indicate higher satisfaction, with previous studies indicating that scores of under 22 indicating dissatisfaction, scores over 22 indicating mild satisfaction, scores over 26 indicating high levels of satisfaction, and scores of over 31 as very high levels of satisfaction (Kelly et al., 2018). The scale has demonstrated adequate psychometric properties in previous studies (Kelly et al., 2018; Larsen et al., 1979).
- **Acceptability Questionnaire (AcQ).** The AQ is a measure of acceptability of remote treatments. The questionnaire has been used in other remote treatments (Wootton et al., 2019a, 2019b).
- **Working Alliance Inventory – Short Form (WAI-SF) (Hatcher & Gillaspy, 2006**). This is a 12-item questionnaire that measures three main aspects of therapeutic alliance: agreement on the tasks of therapy, agreement on the goals of therapy, and development of an emotional bond.
- **Service Use Questionnaire (SUQ).** The SUQ is a measure of any health professionals seen and treatments used by participants due to their anxiety and / or depression symptoms.

The following measure will be administered to all participants at mid-treatment, post-treatment, and follow-up:

- **Adherence Questionnaire (AdQ).** The AQ is a single-item measure of time spent per day working on the skills described in treatment. Participants are asked to describe how many minutes spent per day spent on using the skills presented in treatment.

The following measure will be administered at pre-treatment only:

- **Credibility/Expectancy Questionnaire (CEQ).** The CEQ is a six-item questionnaire measuring client’s expectancy of the treatment and the credibility of the treatment rationale. The questionnaire has demonstrated good test-retest reliability and high internal consistency in previous studies (Devilly & Borkovec, 2000).

The following non-questionnaire, feasibility outcomes will be measured:

- **Participant Retention.** Previous meta-analyses of CBT in the general population have suggested approximately 15.9% of participants drop-out of CBT prior to commencing treatment across disorders, and 26.4% drop out during treatment (Fernandez et al., 2015). This trial will benchmark drop-out rates compared to CBT in the general population, with approximately 80% or higher retention rates after the waitlist and 70% after treatment, indicating preliminary feasibility compared to CBT in the general population. While higher retention rates have been reported in more recent trials of the Unified Protocol (Fujisato et al., 2023; Pachankis et al., 2020), a conservative approach to estimating feasibility has been taken given the preliminary nature of the video-conferencing format of this trial.
- **Recruitment.** Given preliminary power estimates, should at least 52 participants be recruited, then the recruitment methods for CBT delivered via video-conferencing for anxiety disorders will be judged to be feasible. Given power estimates to find clinically meaningful differences in anxiety outcomes, should at least 42 participants be recruited, then the recruitment methods used in this trial may have preliminary feasibility for CBT delivered via video-conferencing for LGBTQ+ adults with anxiety disorders.
- **Questionnaire Completion**. Given power estimates and previous drop out-rates, should sufficient participants complete questionnaires to complete adequately powered analyses, the chosen psychometrics may be suggested to be feasible to be completed via telehealth for LGBTQ+ adults with anxiety and depressive disorders.
- **Treatment Fidelity.** Clinician fidelity to the treatment manuals, as assessed by a supervising clinical psychologist based on treatment manual checklists, will also be assessed. Borrelli et al. (2005) recommended that 80% adherence to treatment guidelines might be assessed as ‘high treatment fidelity’. Supervising clinicians will code fidelity to the treatment manual for 10% of sessions.

All self-report data will be collected via REDCAP. The questionnaire administration schedule is outlined in Table 1 below. Participants in the control group will be re-assessed with the pre-treatment measures and diagnostic interview prior to starting treatment.

**Table 1.**

*Administration Schedule for Outcome Measures*

|  | Screening | Group 1: Immediate Treatment | | | | Group 2: Control Group | | | | |
| --- | --- | --- | --- | --- | --- | --- | --- | --- | --- | --- |
|  | Both groups | Pre-(Week 0) | Mid- | Post-  (Week 9) | 3-month follow-up | Baseline  (Week 0) | Pre-  (Week 9) | Mid- | Post- (Week 18) | 3-month follow-up |
| **Screening Measures** |  |  |  |  |  |  |  |  |  |  |
| *Demographics* | + |  |  |  |  |  |  |  |  |  |
| *Risk questionnaire* | + |  |  |  |  |  |  |  |  |  |
| *DIAMOND Screener* | + |  |  |  |  |  |  |  |  |  |
| *DIAMOND Interview* | + |  |  | + | + |  | + |  | + | + |
| *C-SSRS* | + |  |  |  |  |  |  |  |  |  |
| **Primary Outcome Measures** | | | | | |  | | | | |
| *OASIS* |  | + | + | + | + | + | + | + | + | + |
| **Secondary Outcome** **Measures** | | | | | |  | | | | |
| *ODSIS* |  | + | + | + | + | + | + | + | + | + |
| *GAD-D* |  | + | + | + | + | + | + | + | + | + |
| *SAD-D* |  | + | + | + | + | + | + | + | + | + |
| *PD-D* |  | + | + | + | + | + | + | + | + | + |
| *AG-D* |  | + | + | + | + | + | + | + | + | + |
| *SP-D* |  | + | + | + | + | + | + | + | + | + |
| *SepAD-D* |  | + | + | + | + | + | + | + | + | + |
| *PHQ-9* | + | + | + | + | + | + | + | + | + | + |
| *HHRDS* |  | + | + | + | + | + | + | + | + | + |
| *RSQ-A* |  | + | + | + | + | + | + | + | + | + |
| *LGBIS* |  | + | + | + | + | + | + | + | + | + |
| *SOCS* |  | + | + | + | + | + | + | + | + | + |
| *CGI-S (Self-report)* | + | + | + | + | + | + | + | + | + | + |
| *CGI-I (Self-report)* |  |  | + | + | + |  |  | + | + | + |
| *CGI-S (Assessed)* | + | + | + | + | + | + | + | + | + | + |
| *CGI-I (Assessed)* |  |  | + | + | + |  |  | + | + | + |
| *SUQ* |  |  |  | + | + |  |  |  | + | + |
| *CEQ* |  | + |  |  |  |  | + |  |  |  |
| *AdQ* |  |  | + | + | + |  |  | + | + | + |
| Process Measures |  |  |  |  |  |  |  |  |  |  |
| *CSQ* |  |  | + | + |  |  |  | + | + |  |
| *AcQ* |  |  | + | + |  |  |  | + | + |  |
| *WAI-SF* |  |  | + | + |  |  |  | + | + |  |

*Note*. AcQ = Acceptability Questionnaire; AdQ = Adherence Questionnaire; AG-D = Agoraphobia Dimensional Scale; CEQ = Credibility/Expectancy Questionnaire; CGI-I (Self-report) = NIMH Clinician Global Impression Scale - Impairment; CGI-S (Self-report) = NIMH Clinician Global Impression Scale – Severity; CGI-I (Assessed) = NIMH Clinician Global Impression Scale – Global Improvement, assessed by a clinician; CGI-S (Assessed) = NIMH Clinician Global Impression Scale – Severity (assessed by a clinician); CSQ = Client Satisfaction Questionnaire; C-SSRS = Colombia Suicide Severity Rating Scale; DIAMOND = Diagnostic Interview for Anxiety, Mood, and Obsessive-Compulsive and Related Neuropsychiatric Disorders; DOCS = Dimensional Obsessive Compulsive Scale; GAD-7 = Generalised Anxiety Disorder 7 item measure; HD-D = Hoarding Disorder Dimensional Scale; HHRDS = Heterosexist Harassment, Rejection, and Discrimination Scale; LGBIHS = Lesbian, Gay, Bisexual Identity Scale – Internalised homophobia Subscale; OASIS = Overall Anxiety Severity and Impairment Scale; ODSIS = Overall Depression Severity and Impairment Scale; PD-D = Panic Disorder Dimensional Scale; RSQ-A = Rejection Sensitivity Questionnaire – Adult; SP-D = Specific Phobia Dimensional Scale; SAD-D = Social Anxiety Disorder-Dimensional Scale; PHQ-9 = Patient Health Questionnaire-9 item; SOCS = Sexual Orientation Concealment Scale; SUQ = Service Use Questionnaire; WAI-SF = Working Alliance Inventory – Short Form.

**INCLUSION/EXCLUSION CRITERIA**

The RCT and qualitative semi-structured interviews will utilise the following inclusion criteria:

(1) Presently residing in Australia;

(2) Aged 18 years or over;

(3) Fluent in English;

(4) Meets criteria for an anxiety disorder as either the primary diagnosis or co-occurring with a depressive disorder and the disorder is of at least ‘moderate severity’ (defined as a score of 4 on the DIAMOND module severity measure);

(5) Medication-free or on a stable dose (8 weeks) of psychotropic medication; and

(6) Not presently receiving regular psychological services for their anxiety symptoms (defined as sessions at least once a week with a qualified mental health professional);

(7) Identify as LGBTQ+; and

(8) Have access to a private location to complete the treatment for the duration of the study.

The RCT and qualitative semi-structured interviews will utilise the following exclusion criteria:

(1) Suicide risk as assessed by item 9 of the PHQ-9 (a score of 2 or above) at baseline, or the C-SSRS, or a response of ‘yes’ to item 1-3 of the risk questionnaire, or via clinician judgement during the diagnostic interview.

(2) Engagement in non-suicidal self-injury in the past 12 months;

(2) Daily use of alcohol or illicit drugs (meaning illegal drugs, pharmaceutical drugs used outside of their prescribed or intended use, and other substances inappropriately used);

(3) The presence of a schizophrenia spectrum disorder as assessed by the DIAMOND semi-structured clinical interview;

(4) Any significant cognitive/intellectual impairment as assessed during the diagnostic interview;

(5) A medical condition that may interfere with treatment;

(6) No access to a computer with a camera and stable internet on a regular basis;

(7) Is not willing to engage in treatment on a regular basis using internet-videoconferencing software;

(8) Does not explicitly identify as LGBTQ+;and

(9) Does not indicate any anxiety disorder symptoms on the DIAMOND Self Report screener.

Additionally, participants will need to have attended at least one session of treatment to be invited to the qualitative semi-structured interviews.

Participants without access to stable internet or a private location on a regular basis are unfortunately not eligible to participate in the study due to the infeasibility of providing on-site services in this study (Ojo-Fati et al., 2017). The lack of access to a private place and stable internet creates difficulties for treatment adherence and participant privacy which would require systemic adaption beyond the scope of a feasibility study.

**TIMELINE**

This research program is estimated to continue for 5 years.

**TREATMENT**

Wherever possible, treatment and assessment will be provided by separate therapists. Treatment will be delivered by provisional and registered psychologist(s) under the supervision of a board-approved supervisor on the research team.

Provisional psychologists will be UTS Master of Clinical Psychology students on their clinical placement, meaning that treatment and assessment sessions delivered will count toward their clinical hour count as part of their placement. Before new provisional psychologists begin delivering treatment, an amendment will be submitted to the MREC to advise of the addition. All new clinicians who will provide the treatment will receive a 1–2-hour training session delivered by Mr Dunn. All sessions will be recorded to ensure treatment fidelity, and 10% of these sessions or more will be reviewed by an investigator.

Treatment will be delivered remotely, via videoconferencing on Zoom. Zoom has been selected as the telehealth program of choice due to its compliance with the HIPAA requirements for healthcare (Zoom, 2021) and its availability to the researchers through a University of Technology paid licence with UTS-affiliated information technology and security support. Zoom meets its confidentiality in healthcare needs by encrypting data in motion at the layer of the application using Advanced Encryption Standard (AES) (Zoom, 2021). The clinicians delivering treatment in the study can easily control Zoom privacy settings to prevent unauthorised access by ensuring that all meetings are password protected and using waiting rooms. As meeting hosts, clinicians in the study have the capability to easily remove participants from meetings, which are only available through a privacy-protected link available only to the client and clinician. Participants are informed of and agree to the privacy limitations of accessing treatment in the Participant Information and Consent Form. Previous teleconferencing delivered treatment protocols with ethics approvals have used Zoom as their medium of choice to deliver CBT for anxiety disorders (Trenoska Basile et al., 2022; Winter et al., 2023). Archibald et al. (2019) found that out of 16 healthcare providers qualitatively interviewed via Zoom, most participants rated Zoom more positively than in-person or other videoconferencing platforms due to its security options, cost-effectiveness, and features that support data management.

Treatment will be based on the manualised CBT intervention outlined in the Unified Protocol for the transdiagnostic treatment of internalising disorders (Ellard et al., 2010). The Unified Protocol has been shown to have large effect sizes in previous randomised control trials (Sakiris & Berle, 2019). This treatment has been adapted to be delivered in various contexts in previous clinical trials (Parsons et al., 2017; Wurm et al., 2017), but has not yet been delivered in a video-conferencing format. Despite this, videoconferencing delivered treatment has demonstrated equivalence to standard treatment (Krzyzaniak et al., 2021; Luo et al., 2020) and was largely delivered as standard practice throughout the COVID-19 pandemic.

The immediate treatment group (Group 1) will receive treatment delivered over 50-minute weekly appointments over eight weeks. This treatment is based on the manual for the Unified Protocol (Barlow et al., 2018), and each of the eight modules will be delivered in eight sessions following the assessment sessions of up to two hours. This reflects the Australian psychology community-based context, in which assessment (typically two sessions) and treatment sessions (typically eight sessions) are combined to deliver a total of ten subsidised sessions (Services Australia, 2023). The typical eight-module treatment will cover: psychoeducation on the CBT model and goals; mindfulness of emotions; building cognitive flexibility with restructuring; addressing emotional avoidance and emotion-driven behaviours; behavioural exposures/experiments; and relapse prevention. As in standard CBT practice, participants will be asked to complete tasks in between sessions to improve their therapeutic outcome. A very similar, nine-session adaptation has been used in a previous asynchronous, web-based trial of the Unified Protocol (Tulbure et al., 2018).

After an eight-week waitlist period, the control group will also receive eight weekly 50-minute sessions. This treatment is based on the LGBTQ-Affirmative adapted version of the Unified Protocol (Pachankis et al., 2022). The intervention will still consist of the essential elements of group 1’s treatment but includes adaptions based on previously conducted qualitative interviews with LGBTQ+ stakeholders and therapists with experience working in the LGBTQ+ community (Pachankis et al., 2015). This treatment has been delivered in several randomised control trials (Pachankis et al., 2015; Pachankis et al., 2020), and has demonstrated efficacy. Comparable effect sizes have been found in these previous trials of LGBTQ-adapted CBT as in standard, non-adapted CBT. While there is too little research to conclude equivalence between LGBTQ+ adapted CBT and standard unadapted CBT, these RCT findings suggest potential equivalence given the similar between-group effect sizes found in the general population with standard, unadapted CBT (Hofmann et al., 2012). Participants will be screened for risk at assessment, with risk level higher than low risk indicating exclusion from the study with crisis support services provided to ensure participant safety. This is because the treatment provided in this study is not a crisis service. Additionally, all participants allocated to waitlist control will be provided appropriate resources such as crisis support numbers and an encouragement to engage with their GP or primary care team (See Appendix O). Additionally, participants in the control group are not prohibited from accessing other services.

Further, in a recent survey from late 2022, over 60% of Australian psychologists in the community had stopped accepting new clients or had a waitlist of one month or more, in part due to systemic and workload concerns (Macleod et al., 2023). Thus, unfortunately, it will likely be difficult for participants will be able to access evidence-based psychological treatment free of charge in the community much faster than they would complete their waitlist period, although they are encouraged to contact their GP, use services in the community, including crisis support services, as needed. Receiving free, accessible, and efficacious tailored 1-on-1 CBT treatment available from anywhere in Australia in this study after a 9-week waitlist is likely to be a highly attractive and affordable option for participants.

Each of the modules in the LGBTQ+ adapted treatment protocol will also be delivered in eight sessions to be able to be feasibly delivered in an Australian psychology community-based context (Services Australia, 2023). The typical treatment will cover psychoeducation on the CBT model and goals; understanding the nature and emotional impact of LGBTQ-​related stress; mindfulness of emotions; building cognitive flexibility with restructuring; addressing emotional behaviours; behavioural exposures/experiments; and relapse prevention. As in standard CBT practice, participants will be asked to complete tasks in between sessions to improve their therapeutic outcome.

At post-treatment and three-month follow-up, all participants will receive feedback on their progress as measured by psychometric outcome measures either in writing via email or in person if requested. An invitation to participate in the interview will be attached with the PCIF. Participants who drop-out or do not respond to follow-up will be encouraged to consult with their General Practitioner who can refer on to local treatment options.

The qualitative semi-structured interview will be conducted by the student researcher, ID, and recorded for transcription via NVIVO.

**TRIAL REGISTRATION**

The RCT will be registered with the Australian and New Zealand Clinical Trials Register (ANZCTR) prior to commencing treatment. The study protocol will also be published.

**DATA STORAGE**

Participant data will be de-identified and collected under ethics approval guidelines. Data will be collected and stored for these studies in methods consistent with the guidelines for human research (NHMRC, 2019). Study 1’s Research Data Management Plan via STASH is included in the Appendix Q. Clinical trial data will be stored for 15 years (NHMRC, 2019) on campus in a locked facility, on OneDrive, and on the UTS-provided eResearch platform.

Recordings of interviews will be stored on an encrypted UTS CloudStor folder and deleted from the original source. At the completion of data analysis, interview data will be transferred to a secure eResearch fileshare via UTS’s servers. This will be able to be accessed by the research team only. During the phase of data analysis, non-identifiable data will be used that excludes information such as names and specific addresses. Once the study is complete, non-identifiable data will be written to a password-protected file.

**ANALYSIS PLAN**

To meet the study’s aim (i.e., to understand if the treatment is efficacious for reducing symptoms of anxiety disorders ), the following analyses will be conducted. The main analyses looking at treatment outcomes from the RCT will be carried out using conservative intention-to-treat principles and using mixed-linear models analyses to handle missing data. Mixed-models are a robust statistical approach for analysing longitudinal clinical trial data and these analyses will employ an appropriate covariance structure and maximum likelihood estimation, which provides unbiased estimates in the case of missing data, under the assumption that data is missing at random. These analyses account for the effect of missing data, thus additional participant recruitment to account for drop-out rates is not warranted. Using this dataset, other appropriate analyses arising from secondary papers will also be conducted. Participants have been informed in the PICF that other secondary data analyses will be conducted on the data that they provide during their participation in the research trial. This may include intersectional moderator analyses of treatment efficacy based on person-level stigma moderators from the minority stress model to inform further treatment adaption needs (Barrera & Castro, 2006; Meyer, 2003). Comparisons between the standard treatment and the LGBTQ-affirmative CBT protocol will be compared using benchmarking analyses (Minami et al., 2008).

To understand the treatment’s efficacy, clinically significant change will be measured using three methods. Diagnostic change will be assessed from pre- to post-treatment and at follow-up using the DIAMOND structured interview. Treatment response will be measured using the reliable change index (RCI) of Jacobson and Truax (1991) to understand which change on the OASIS is not attributable to measurement error, but rather is statistically reliable. Any increase in symptoms by the RCI magnitude of change will be defined as deterioration in this trial. Clinically significant change at post-treatment and to follow-up will be defined as a score below the cut score of 8 on the OASIS (Campbell-Sills et al., 2009).

To meet the study’s aim to quantitatively understand if the treatment is feasible and acceptable for LGBTQ+ participants, proportions will be calculated for participant drop-out, questionnaire completion, recruitment, and treatment fidelity. Treatment fidelity will be calculated by summing for each session the total number of treatment components which were coded as ‘present’, divided by the total applicable number of treatment component items (Borrelli et al., 2005). Results will be benchmarked with previous estimates of CBT in the general population and for the Unified Protocol.

To meet the study’s aim to qualitatively understand if the treatment is feasible and acceptable for LGBTQ+ participants, thematic analysis of the qualitative interviews will be conducted in NVivo12 using framework analysis (Ritchie & Spencer, 1994). Six phases will be followed. Firstly, interview transcripts will be read and re-read to understand the data content. Secondly, each transcript will be coded to capture the meaning of participants’ experience in a condensed way. These will be discussed with the research team to cross-check the codes. Across the interviews, codes will then be collapsed. Fourthly, using a thematic map, codes will be sorted into different themes to provide an overview. Fifthly, themes will be refined by checking their coherence with original codes. Lastly, final themes will be decided and named, with production of the analysis report. Selected quotes around causes for treatment non-effectiveness or treatment effectiveness will be reported, with abbreviation if meaning is not affected, to help contextualise quantitative results of treatment efficacy.

To ensure rigour in the qualitative methodology, the model of trustworthiness proposed by Lincoln and Guba (1985) will be used. Lincoln and Guba recommended that researchers can indicate trustworthy findings by demonstrating their research's credibility, transferability, dependability, and confirmability. Recommendations for embedding these criteria in reflexive thematic analyses were made by Nowell et al. (2017), which include: prolonged data engagement; reflexive journaling (i.e., memos); well-organized storage of data, field notes, memos and related documents; debriefing with peers; for referential adequacy of candidate themes, returning to raw data, and; providing detailed description of the research context and an audit trail. Each of these recommendations was included in this studies’ design.

**REPORTING OF ADVERSE EVENTS**

Regular team meetings are conducted between the team and treatment integrity is monitored by registered psychologists. Participants are monitored regularly throughout treatment during their treatment sessions and via self-report questionnaires, available in Appendix P. The Chief Investigator and Student Investigator have completed the good clinical practice training at UTS.

As this study is not trialling the effects of a pharmaceutical drug or medical equipment but rather the effects of talk therapy, potential adverse events to endanger participant safety are rare and unknown (Duggan et al., 2014). As such, no significant safety issues (SSI) or serious adverse events (SAEs) are expected in this study, but may still occur, and so a log will be kept of all SSIs or SAEs that arise including their potential relatedness to the treatment. In all adverse events, the treating clinician will take steps to ensure the participant’s immediate health and safety using clinical judgment in collaboration with the principal investigator. SAEs in this study are defined as hospitalisation, death, adverse events resulting in permanent harm or disability, or life-threatening or otherwise medically significant risks as assessed by the clinician. When detected, SAEs will be reported to the Health and Medical Research Ethics Committee by the principal investigator within 24 hours along with details of the event and actions taken to mitigate risk from the adverse events log. SSIs will be defined as marked deterioration in symptoms measured by a decrease in self-report questionnaires of a magnitude denoted by the reliable change index (Jacobson & Truax, 1991), clinician clinical judgement, or a combination of both. Once detected, participant risk will be assessed as is standard in clinical practice. Participants whose risk level escalates or whose symptoms markedly deteriorate will be encouraged to contact their health professional team, such as their GP, or emergency services, and refer to their safety plan made at the beginning of treatment.

**FUNDING**

The student investigator is partially funded by the Department of Education to complete their research under a Research Training Program (RTP) Stipend. There are no further conflicts of interest to report, as this is an unfunded study. The MREC will be notified via an amendment should future funding be obtained to support this research.

**COMMUNICATION WITH HEALTH PROFESSIONALS**

Participants will be asked in their pre-treatment, post-treatment, and follow-up questionnaires if they would like a letter sent to their treating health professional(s) outlining their participation in the study (pre-treatment) and, at post-treatment and follow-up, their pre-treatment, post-treatment, and follow-up outcome scores on the OASIS. To consent, participants will be asked to tick a box and to provide the name and postal details of their health professional(s). Those who are interested will be sent a template letter providing this information at pre-treatment, post-treatment, and follow-up (see Appendix N).

**References**

American Psychological Association Presidential Task Force on Evidence-Based Practice. (2006). Evidence-based practice in psychology. *The American Psychologist*, *61*(4), 271-285. <https://doi.org/10.1037/0003-066X.61.4.271>

Archibald, M. M., Ambagtsheer, R. C., Casey, M. G., & Lawless, M. (2019). Using Zoom Videoconferencing for Qualitative Data Collection: Perceptions and Experiences of Researchers and Participants. *International Journal of Qualitative Methods*, *18*, 1609406919874596. <https://doi.org/10.1177/1609406919874596>

Australian Bureau of Statistics. (2023). *National Study of Mental Health and Wellbeing 2020-2022*. Retrieved from <https://www.abs.gov.au/statistics/health/mental-health/national-study-mental-health-and-wellbeing/latest-release#:~:text=Prevalence%20of%2012%2Dmonth%20mental,a%2012%2Dmonth%20Affective%20disorder>

Australian Psychological Society. (2018). *Evidence-based psychological interventions in the treatment of mental disorders: A review of the literature*. Retrieved from <https://psychology.org.au/getmedia/23c6a11b-2600-4e19-9a1d-6ff9c2f26fae/evidence-based-psych-interventions.pdf>

Barlow, D. H., Sauer-Zavala, S., Farchione, T. J., Latin, H. M., Ellard, K. K., Bullis, J. R., Bentley, K. H., Boettcher, H. T., & Cassiello-Robbins, C. (2018). *Unified protocol for transdiagnostic treatment of emotional disorders: workbook* (Second edition ed.). New York, NY.

Barrera, M., & Castro, F. G. (2006). A heuristic framework for the cultural adaptation of interventions. *Clinical Psychology: Science and Practice*, *13*(4), 311-316. <https://doi.org/10.1111/j.1468-2850.2006.00043.x>

Bentley, K. H., Gallagher, M. W., Carl, J. R., & Barlow, D. H. (2014). Development and validation of the Overall Depression Severity and Impairment Scale. *Psychological assessment*, *26*(3), 815-830. <https://doi.org/10.1037/a0036216>

Berenson, K. R., Gyurak, A., Ayduk, O., Downey, G., Garner, M. J., Mogg, K., Bradley, B. P., & Pine, D. S. (2009). Rejection sensitivity and disruption of attention by social threat cues. *J Res Pers*, *43*(6), 1064-1072. <https://doi.org/10.1016/j.jrp.2009.07.007>

Borrelli, B., Sepinwall, D., Ernst, D., Bellg, A. J., Czajkowski, S., Breger, R., DeFrancesco, C., Levesque, C., Sharp, D. L., Ogedegbe, G., Resnick, B., & Orwig, D. (2005). A new tool to assess treatment fidelity and evaluation of treatment fidelity across 10 years of health behavior research. *J Consult Clin Psychol*, *73*(5), 852-860. <https://doi.org/10.1037/0022-006x.73.5.852>

Braun, V., & Clarke, V. (2013). Successful qualitative research: A practical guide for beginners. *Successful qualitative research*, 1-400.

Campbell-Sills, L., Norman, S. B., Craske, M. G., Sullivan, G., Lang, A. J., Chavira, D. A., Bystritsky, A., Sherbourne, C., Roy-Byrne, P., & Stein, M. B. (2009). Validation of a brief measure of anxiety-related severity and impairment: the Overall Anxiety Severity and Impairment Scale (OASIS). *J Affect Disord*, *112*(1-3), 92-101. <https://doi.org/10.1016/j.jad.2008.03.014>

Christensen, I., Power, E., Togher, L., & Norup, A. (2023). "Communication Is Not Exactly My Field, but It Is Still My Area of Work": Staff and Managers' Experiences of Communication With People With Traumatic Brain Injury. *American journal of speech-language pathology*, *32*(2S), 827-847. <https://doi.org/10.1044/2022_AJSLP-22-00074>

ClinCalc LLC. (2024). *Sample Size Calculator*. Retrieved 2 February 2024 from <https://clincalc.com/Stats/SampleSize.aspx>

Cuijpers, P., Berking, M., Andersson, G., Quigley, L., Kleiboer, A., & Dobson, K. S. (2013). A meta-analysis of cognitive-behavioural therapy for adult depression, alone and in comparison with other treatments. *The Canadian Journal of Psychiatry*, *58*(7), 376-385. <https://doi.org/10.1177/070674371305800702>

Devilly, G. J., & Borkovec, T. D. (2000). Psychometric properties of the credibility/expectancy questionnaire. *Journal of Behavior Therapy and Experimental Psychiatry*, *31*(2), 73-86. <https://doi.org/https://doi.org/10.1016/S0005-7916(00)00012-4>

Duggan, C., Parry, G., McMurran, M., Davidson, K., & Dennis, J. (2014). The recording of adverse events from psychological treatments in clinical trials: evidence from a review of NIHR-funded trials. *Trials*, *15*, 335. <https://doi.org/10.1186/1745-6215-15-335>

Ellard, K. K., Fairholme, C. P., Boisseau, C. L., Farchione, T. J., & Barlow, D. H. (2010). Unified Protocol for the transdiagnostic treatment of emotional disorders: Protocol development and initial outcome data. *Cognitive and behavioral practice*, *17*(1), 88-101. <https://doi.org/10.1016/j.cbpra.2009.06.002>

Faul, F., Erdfelder, E., Lang, A.-G., & Buchner, A. (2007). G*Power 3: A flexible statistical power analysis program for the social, behavioral, and biomedical sciences. *Behavior Research Methods*, *39*(2), 175-191. <https://doi.org/10.3758/BF03193146>

Fernandez, E., Salem, D., Swift, J. K., & Ramtahal, N. (2015). Meta-analysis of dropout from cognitive behavioral therapy: Magnitude, timing, and moderators. *Journal of Consulting and Clinical Psychology*, *83*(6), 1108-1122. <https://doi.org/10.1037/ccp0000044>

Foy, A. A. J., Morris, D., Fernandes, V., & Rimes, K. A. (2019). LGBQ+ adults’ experiences of Improving Access to Psychological Therapies and primary care counselling services: informing clinical practice and service delivery. *the Cognitive Behaviour Therapist*, *12*, e42, Article e42. <https://doi.org/10.1017/S1754470X19000291>

Fujisato, H., Horikoshi, M., Ito, M., Kato, N., Miyamae, M., Nakajima, S., Oe, Y., Okumura, Y., Takebayashi, Y., Toyota, A., & Yamaguchi, K. (2023). Efficacy of the unified protocol for transdiagnostic cognitive-behavioral treatment for depressive and anxiety disorders: A randomized controlled trial. *Psychological Medicine*, *53*(7), 3009-3020. <https://doi.org/10.1017/S0033291721005067>

Greenberg, P. E., Fournier, A. A., Sisitsky, T., Simes, M., Berman, R., Koenigsberg, S. H., & Kessler, R. C. (2021). The economic burden of adults with major depressive disorder in the United States (2010 and 2018). *PharmacoEconomics*, *39*(6), 653-665. <https://doi.org/10.1007/s40273-021-01019-4>

Guy, W. (1976). ECDEU Assessment Manual for Psychopharmacology. In E. Department of Health, and Welfare (Ed.).

Hall, W. J., Rosado, B. R., & Chapman, M. V. (2019). Findings from a Feasibility Study of an Adapted Cognitive Behavioral Therapy Group Intervention to Reduce Depression among LGBTQ (Lesbian, Gay, Bisexual, Transgender, or Queer) Young People. *J Clin Med*, *8*(7). <https://doi.org/10.3390/jcm8070949>

Hatzenbuehler, M. L. (2009). How does sexual minority stigma "get under the skin"? A psychological mediation framework. *Psychological Bulletin*, *135*(5), 707-730. <https://doi.org/10.1037/a0016441>

Hinshaw, S. P., & Stier, A. (2008). Stigma as related to mental disorders. *Annual Review of Clinical Psychology*, *4*(1), 367-393. <https://doi.org/10.1146/annurev.clinpsy.4.022007.141245>

Hofmann, S. G., Asnaani, A., Vonk, I. J. J., Sawyer, A. T., & Fang, A. (2012). The efficacy of cognitive behavioral therapy: A review of meta-analyses. *Cognitive Therapy and Research*, *36*(5), 427-440. <https://doi.org/10.1007/s10608-012-9476-1>

Jackson, S. D., & Mohr, J. J. (2016). *Conceptualizing the closet: Differentiating stigma concealment and nondisclosure processes* [doi:10.1037/sgd0000147]. Educational Publishing Foundation.

Jackson, S. D., Wagner, K. R., Yepes, M., Harvey, T. D., Higginbottom, J., & Pachankis, J. E. (2022). A pilot test of a treatment to address intersectional stigma, mental health, and HIV risk among gay and bisexual men of color. *Psychotherapy*, *59*(1), 96-112. <https://doi.org/10.1037/pst0000417>

Jacobson, N. S., & Truax, P. (1991). Clinical significance: a statistical approach to defining meaningful change in psychotherapy research. *Journal of Consulting and Clinical Psychology*, *59*(1), 12-19. <https://doi.org/10.1037//0022-006x.59.1.12>

Kalev, A., Dobbin, F., & Kelly, E. (2006). Best practices or best guesses? Assessing the efficacy of corporate affirmative action and diversity policies. *American Sociological Review*, *71*(4), 589-617. <https://doi.org/10.1177/000312240607100404>

Kelly, P. J., Kyngdon, F., Ingram, I., Deane, F. P., Baker, A. L., & Osborne, B. A. (2018). The Client Satisfaction Questionnaire-8: Psychometric properties in a cross-sectional survey of people attending residential substance abuse treatment. *Drug and Alcohol Review*, *37*(1), 79-86. <https://doi.org/https://doi.org/10.1111/dar.12522>

Kessler, R. C., Ormel, J., Petukhova, M., McLaughlin, K. A., Green, J. G., Russo, L. J., Stein, D. J., Zaslavsky, A. M., Aguilar-Gaxiola, S., Alonso, J., Andrade, L., Benjet, C., de Girolamo, G., de Graaf, R., Demyttenaere, K., Fayyad, J., Haro, J. M., Hu, C. y., Karam, A., . . . Üstün, T. B. (2011). Development of lifetime comorbidity in the World Health Organization world mental health surveys. *Archives of General Psychiatry*, *68*(1), 90-100. <https://doi.org/10.1001/archgenpsychiatry.2010.180>

Konnopka, A., & König, H. (2020). Economic burden of anxiety disorders: A systematic review and meta‑analysis. *PharmacoEconomics*, *38*(1), 25-37. <https://doi.org/https://doi.org/10.1007/s40273-019-00849-7>

Kroenke, K., Spitzer, R. L., & Williams, J. B. W. (2001). The PHQ-9: Validity of a brief depression severity measure. *Journal of General Internal Medicine*, *16*(9), 606-613. <https://doi.org/10.1046/j.1525-1497.2001.016009606.x>

Krueger, R. F. (1999). The structure of common mental disorders. *Archives of General Psychiatry*, *56*(10), 921 - 926. <https://doi.org/10.1001/archpsyc.56.10.921>

Krzyzaniak, N., Greenwood, H., Scott, A. M., Peiris, R., Cardona, M., Clark, J., & Glasziou, P. (2021). The effectiveness of telehealth versus face-to face interventions for anxiety disorders: A systematic review and meta-analysis. *Journal of Telemedicine and Telecare*, *0*(0), 1357633X211053738. <https://doi.org/10.1177/1357633x211053738>

Larsen, D. L., Attkisson, C. C., Hargreaves, W. A., & Nguyen, T. D. (1979). Assessment of client/patient satisfaction: Development of a general scale [Article]. *Evaluation and Program Planning*, *2*(3), 197-207. <https://doi.org/10.1016/0149-7189(79)90094-6>

Lebeau, R. T., Glenn, D. E., Hanover, L. N., Beesdo-Baum, K., Wittchen, H. U., & Craske, M. G. (2012). A dimensional approach to measuring anxiety for DSM-5. *Int J Methods Psychiatr Res*, *21*(4), 258-272. <https://doi.org/10.1002/mpr.1369>

Leon, A. C., Shear, M. K., Portera, L., & Klerman, G. L. (1992). Assessing impairment in patients with panic disorder: the Sheehan Disability Scale [Article]. *Social Psychiatry and Psychiatric Epidemiology*, *27*(2), 78-82. <https://doi.org/10.1007/BF00788510>

Lincoln, Y. S., & Guba, E. G. (1985). *Naturalistic inquiry*. sage.

Luo, C., Sanger, N., Singhal, N., Pattrick, K., Shams, I., Shahid, H., Hoang, P., Schmidt, J., Lee, J., Haber, S., Puckering, M., Buchanan, N., Lee, P., Ng, K., Sun, S., Kheyson, S., Chung, D. C.-Y., Sanger, S., Thabane, L., & Samaan, Z. (2020). A comparison of electronically-delivered and face to face cognitive behavioural therapies in depressive disorders: A systematic review and meta-analysis. *eClinicalMedicine*, *24*. <https://doi.org/10.1016/j.eclinm.2020.100442>

Macleod, E., Curll, S., Walker, I., Reynolds, J., Lane, J., Galati, C., Greenwood, L., Christensen, B., & Calear, A. L. (2023). *Australian psychologists in the context of disasters: Preliminary report on workforce impacts and needs*.

Malterud, K., Siersma, V. D., & Guassora, A. D. (2016). Sample size in qualitative interview studies: Guided by information power. *Qualitative Health Research*, *26*(13), 1753-1760. <https://doi.org/10.1177/1049732315617444>

Manea, L., Gilbody, S., & McMillan, D. (2012). Optimal cut-off score for diagnosing depression with the Patient Health Questionnaire (PHQ-9): A meta-analysis [Article]. *CMAJ*, *184*(3), E191-E196. <https://doi.org/10.1503/cmaj.110829>

Matsumoto, K., Hamatani, S., & Shimizu, E. (2021). Effectiveness of Videoconference-Delivered Cognitive Behavioral Therapy for Adults With Psychiatric Disorders: Systematic and Meta-Analytic Review. *J Med Internet Res*, *23*(12), e31293. <https://doi.org/10.2196/31293>

McGrath, J. J., Al-Hamzawi, A., Alonso, J., Altwaijri, Y., Andrade, L. H., Bromet, E. J., Bruffaerts, R., de Almeida, J. M. C., Chardoul, S., Chiu, W. T., Degenhardt, L., Demler, O. V., Ferry, F., Gureje, O., Haro, J. M., Karam, E. G., Karam, G., Khaled, S. M., Kovess-Masfety, V., . . . Collaborators, W. H. O. W. M. H. S. (2023). Age of onset and cumulative risk of mental disorders: a cross-national analysis of population surveys from 29 countries. *Lancet Psychiatry*, *10*(9), 668-681. <https://doi.org/10.1016/S2215-0366(23)00193-1>

Meyer, I. H. (2003). Prejudice, social stress, and mental health in lesbian, gay, and bisexual populations: conceptual issues and research evidence. *Psychological Bulletin*, *129*(5), 674-697. <https://doi.org/10.1037/0033-2909.129.5.674>

Minami, T., Serlin, R. C., Wampold, B. E., Kircher, J. C., & Brown, G. S. (2008). Using clinical trials to benchmark effects produced in clinical practice. *Quality and Quantity*, *42*(4), 513-525. <https://doi.org/10.1007/s11135-006-9057-z>

Mira, A., González-Robles, A., Molinari, G., Miguel, C., Díaz-García, A., Bretón-López, J., García-Palacios, A., Quero, S., Baños, R., & Botella, C. (2019). Capturing the Severity and Impairment Associated With Depression: The Overall Depression Severity and Impairment Scale (ODSIS) Validation in a Spanish Clinical Sample [Original Research]. *Frontiers in Psychiatry*, *10*. <https://doi.org/10.3389/fpsyt.2019.00180>

Mohr, J. J., & Kendra, M. S. (2011). Revision and Extension of a Multidimensional Measure of Sexual Minority Identity: The Lesbian, Gay, and Bisexual Identity Scale. *Journal of counseling psychology*, *58*(2), 234-245. <https://doi.org/10.1037/a0022858>

National Institute for Health and Care Excellence. (2011). *Common mental health problems: identification and pathways to care*. Retrieved from <https://www.nice.org.uk/guidance/cg123/chapter/Recommendations>

Norman, S. B., Hami Cissell, S., Means-Christensen, A. J., & Stein, M. B. (2006). Development and validation of an Overall Anxiety Severity And Impairment Scale (OASIS). *Depression and Anxiety*, *23*(4), 245-249. <https://doi.org/10.1002/da.20182>

Nowell, L. S., Norris, J. M., White, D. E., & Moules, N. J. (2017). Thematic Analysis: Striving to Meet the Trustworthiness Criteria. *International Journal of Qualitative Methods*, *16*(1), 1609406917733847. <https://doi.org/10.1177/1609406917733847>

Ojo-Fati, O., Joseph, A. M., Ig-Izevbekhai, J., Thomas, J. L., Everson-Rose, S. A., Pratt, R., Raymond, N., Cooney, N. L., Luo, X., & Okuyemi, K. S. (2017). Practical issues regarding implementing a randomized clinical trial in a homeless population: strategies and lessons learned. *Trials*, *18*(1), 305. <https://doi.org/10.1186/s13063-017-2046-9>

Olfson, M., Guardino, M., Struening, E., Schneier, F. R., Hellman, F., & Klein, D. F. (2000). Barriers to the Treatment of Social Anxiety. *American Journal of Psychiatry*, *157*(4), 521-527. <https://doi.org/10.1176/appi.ajp.157.4.521>

Pachankis, J. E. (2014). Uncovering Clinical Principles and Techniques to Address Minority Stress, Mental Health, and Related Health Risks Among Gay and Bisexual Men. *Clinical psychology (New York, N.Y.)*, *21*(4), 313-330. <https://doi.org/10.1111/cpsp.12078>

Pachankis, J. E., Harkness, A., Jackson, S., & Safren, S. A. (2022). *Transdiagnostic LGBTQ-Affirmative Cognitive-Behavioral Therapy: Therapist Guide*. Oxford University Press, Incorporated.

Pachankis, J. E., Hatzenbuehler, M. L., Rendina, H. J., Safren, S. A., & Parsons, J. T. (2015). LGB-affirmative cognitive-behavioral therapy for young adult gay and bisexual men: A randomized controlled trial of a transdiagnostic minority stress approach. *Journal of Consulting and Clinical Psychology*, *83*(5), 875-889. <https://doi.org/10.1037/ccp0000037>

Pachankis, J. E., McConocha, E. M., Clark, K. A., Wang, K., Behari, K., Fetzner, B. K., Brisbin, C. D., Scheer, J. R., & Lehavot, K. (2020). A transdiagnostic minority stress intervention for gender diverse sexual minority women's depression, anxiety, and unhealthy alcohol use: A randomized controlled trial. *Journal of Consulting and Clinical Psychology*, *88*(7), 613-630. <https://doi.org/10.1037/ccp0000508>

Pachankis, J. E., Soulliard, Z. A., Layland, E. K., Behari, K., Seager van Dyk, I., Eisenstadt, B. E., Chiaramonte, D., Ljótsson, B., Särnholm, J., & Bjureberg, J. (2023). Guided LGBTQ-affirmative internet cognitive-behavioral therapy for sexual minority youth's mental health: A randomized controlled trial of a minority stress treatment approach. *Behaviour Research and Therapy*, *169*, 104403. <https://doi.org/https://doi.org/10.1016/j.brat.2023.104403>

Parsons, J. T., Rendina, H. J., Moody, R. L., Gurung, S., Starks, T. J., & Pachankis, J. E. (2017). Feasibility of an Emotion Regulation Intervention to Improve Mental Health and Reduce HIV Transmission Risk Behaviors for HIV-Positive Gay and Bisexual Men with Sexual Compulsivity. *AIDS Behav*, *21*(6), 1540-1549. <https://doi.org/10.1007/s10461-016-1533-4>

Patton, G. C., Coffey, C., Romaniuk, H., Mackinnon, A., Carlin, J. B., Degenhardt, L., Olsson, C. A., & Moran, P. (2014). The prognosis of common mental disorders in adolescents: a 14-year prospective cohort study. *The Lancet*, *383*(9926), 1404-1411. <https://doi.org/10.1016/S0140-6736(13)62116-9>

Posner, K., Brent, D., Lucas, C., Gould, M., Stanley, B., Brown, G., Fisher, P., Zelazny, J., Burke, A., & Oquendo, M. (2008). Columbia-suicide severity rating scale (C-SSRS). *New York, NY: Columbia University Medical Center*, *10*, 2008.

Reinecke, A., Thilo, K. V., Croft, A., & Harmer, C. J. (2018). Early effects of exposure-based cognitive behaviour therapy on the neural correlates of anxiety. *Translational Psychiatry*, *8*(1), 225. <https://doi.org/10.1038/s41398-018-0277-5>

Ritchie, J., & Spencer, L. (1994). Qualitative data analysis for applied policy research. In A. Bryman & B. Burgess (Eds.), *Analyzing Qualitative Data*. Taylor & Francis Group. <http://ebookcentral.proquest.com/lib/uts/detail.action?docID=170016>

Sakiris, N., & Berle, D. (2019). A systematic review and meta-analysis of the Unified Protocol as a transdiagnostic emotion regulation based intervention. *Clinical Psychology Review*, *72*, 101751. <https://doi.org/10.1016/j.cpr.2019.101751>

Scott, A. J., Bisby, M. A., Heriseanu, A. I., Hathway, T., Karin, E., Gandy, M., Dudeney, J., Staples, L. G., Titov, N., & Dear, B. F. (2022). Understanding the untreated course of anxiety disorders in treatment-seeking samples: A systematic review and meta-analysis. *Journal of Anxiety Disorders*, *89*, 102590. <https://doi.org/https://doi.org/10.1016/j.janxdis.2022.102590>

Sekhon, M., Cartwright, M., & Francis, J. J. (2017). Acceptability of healthcare interventions: an overview of reviews and development of a theoretical framework. *BMC Health Services Research*, *17*(1), 88. <https://doi.org/10.1186/s12913-017-2031-8>

Services Australia. (2023). *Mental health care and Medicare*. Retrieved 8 November 2023 from <https://www.servicesaustralia.gov.au/mental-health-care-and-medicare?context=60092>

Sheehan, D. V. (1983). *The anxiety disease*. Scribner.

Spijker, J., de Graaf, R., Bijl, R. V., Beekman, A. T., Ormel, J., & Nolen, W. A. (2002). Duration of major depressive episodes in the general population: results from The Netherlands Mental Health Survey and Incidence Study (NEMESIS). *Br J Psychiatry*, *181*, 208-213. <https://doi.org/10.1192/bjp.181.3.208>

Springer, K. S., Levy, H. C., & Tolin, D. F. (2018). Remission in CBT for adult anxiety disorders: A meta-analysis. *Clin Psychol Rev*, *61*, 1-8. <https://doi.org/10.1016/j.cpr.2018.03.002>

Szymanski, D. M. (2006). Does Internalized Heterosexism Moderate the Link Between Heterosexist Events and Lesbians' Psychological Distress? *Sex Roles*, *54*(3), 227-234. <https://doi.org/10.1007/s11199-006-9340-4>

Tolin, D. F., Gilliam, C., Wootton, B. M., Bowe, W., Bragdon, L. B., Davis, E., Hannan, S. E., Steinman, S. A., Worden, B., & Hallion, L. S. (2018). Psychometric properties of a structured diagnostic interview for DSM-5 anxiety, mood, and obsessive-compulsive and related disorders [Article]. *Assessment*, *25*(1), 3-13. <https://doi.org/10.1177/1073191116638410>

Tong, A., Sainsbury, P., & Craig, J. (2007). Consolidated criteria for reporting qualitative research (COREQ): a 32-item checklist for interviews and focus groups. *International Journal for Quality in Health Care*, *19*(6), 349-357. <https://doi.org/10.1093/intqhc/mzm042>

Trenoska Basile, V., Newton-John, T., & Wootton, B. M. (2022). Internet videoconferencing delivered cognitive behavior therapy for generalized anxiety disorder: protocol for a randomized controlled trial. *Trials*, *23*(1), 592. <https://doi.org/10.1186/s13063-022-06520-5>

Tulbure, B. T., Rusu, A., Sava, F. A., Sălăgean, N., & Farchione, T. J. (2018). A Web-Based Transdiagnostic Intervention for Affective and Mood Disorders: Randomized Controlled Trial. *JMIR Ment Health*, *5*(2), e36. <https://doi.org/10.2196/mental.8901>

Van Der Pol-Harney, E., & McAloon, J. (2018). Psychosocial Interventions for mental illness among LGBTQIA youth: A PRISMA-based systematic review. *Adolescent Research Review*, *4*(2), 149-168. <https://doi.org/10.1007/s40894-018-0090-7>

Winter, H. R., Norton, A., & Wootton, B. M. (2023). Internet videoconferencing delivered cognitive behavioral therapy for social anxiety disoder: Protocol for a randomized controlled trial. *Contempary Clinical Trials*, *132*, 107298. <https://doi.org/10.1016/j.cct.2023.107298>

Wootton, B. M., Karin, E., Titov, N., & Dear, B. F. (2019a). Self-Guided Internet Delivered Cognitive Behavior Therapy (ICBT) for Obsessive-Compulsive Symptoms: A Randomized Controlled Trial. *Journal of Anxiety Disorders*(66), 102111. <https://doi.org/https://doi.org/10.1016/j.janxdis.2019.102111>

Wootton, B. M., Karin, E., Titov, N., & Dear, B. F. (2019b). Self-guided internet–delivered cognitive behavior therapy (ICBT) for obsessive-compulsive symptoms: A randomized controlled trial [Article]. *Journal of Anxiety Disorders*, *66*, Article 102111. <https://doi.org/10.1016/j.janxdis.2019.102111>

Wurm, M., Klein Strandberg, E., Lorenz, C., Tillfors, M., Buhrman, M., Holländare, F., & Boersma, K. (2017). Internet delivered transdiagnostic treatment with telephone support for pain patients with emotional comorbidity: a replicated single case study. *Internet Interventions*, *10*, 54-64. <https://doi.org/https://doi.org/10.1016/j.invent.2017.10.004>

Zoom. (2021). HIPAA Compliance Datasheet. In Zoom (Ed.).

Zuithoff, N. P., Vergouwe, Y., King, M., Nazareth, I., Van Wezep, M. J., Moons, K. G., & Geerlings, M. I. (2010). The patient health questionnaire-9 for detection of major depressive disorder in primary care: Consequences of current thresholds in a crosssectional study [Article]. *BMC Family Practice*, *11*, Article 98. <https://doi.org/10.1186/1471-2296-11-98>
